# De novo assembly of the olive fruit fly (*Bactrocera oleae*) genome with linked-reads and long-read technologies minimizes gaps and provides exceptional Y chromosome assembly

**DOI:** 10.1186/s12864-020-6672-3

**Published:** 2020-03-30

**Authors:** Anthony Bayega, Haig Djambazian, Konstantina T. Tsoumani, Maria-Eleni Gregoriou, Efthimia Sagri, Eleni Drosopoulou, Penelope Mavragani-Tsipidou, Kristina Giorda, George Tsiamis, Kostas Bourtzis, Spyridon Oikonomopoulos, Ken Dewar, Deanna M. Church, Alexie Papanicolaou, Kostas D. Mathiopoulos, Jiannis Ragoussis

**Affiliations:** 1 McGill University and Genome Quebec Innovation Centre, Department of Human Genetics, McGill University, Montreal, Canada; 2 Department of Biochemistry and Biotechnology, University of Thessaly, Biopolis, 41500 Larissa, Greece; 3 Department of Biology, Aristotle University of Thessaloniki, Thessaloniki, Greece; 4 Integrated DNA Technologies, Inc., 1710 Commercial Park, Coralville, Iowa, 52241 USA; 5 Department of Environmental Engineering, University of Patras, Agrinio, Greece; 6 Insect Pest Control Laboratory, Joint FAO/IAEA Division of Nuclear Techniques in Food and Agriculture, Vienna, Austria; 7 Inscripta, Inc., 5500 Central Avenue #220, Boulder, CO 80301 USA; 8 Hawkesbury Institute for the Environment, Western Sydney University, Richmond, NSW 2753 Australia

**Keywords:** Olive fruit fly genome, *Bactrocera oleae*, Linked reads, Long reads, Y chromosome assembly, Insect developmental genes

## Abstract

**Background:**

The olive fruit fly, *Bactrocera oleae*, is the most important pest in the olive fruit agribusiness industry. This is because female flies lay their eggs in the unripe fruits and upon hatching the larvae feed on the fruits thus destroying them. The lack of a high-quality genome and other genomic and transcriptomic data has hindered progress in understanding the fly’s biology and proposing alternative control methods to pesticide use.

**Results:**

Genomic DNA was sequenced from male and female Demokritos strain flies, maintained in the laboratory for over 45 years. We used short-, mate-pair-, and long-read sequencing technologies to generate a combined male-female genome assembly (GenBank accession GCA_001188975.2). Genomic DNA sequencing from male insects using 10x Genomics linked-reads technology followed by mate-pair and long-read scaffolding and gap-closing generated a highly contiguous 489 Mb genome with a scaffold N50 of 4.69 Mb and L50 of 30 scaffolds (GenBank accession GCA_001188975.4). RNA-seq data generated from 12 tissues and/or developmental stages allowed for genome annotation. Short reads from both males and females and the chromosome quotient method enabled identification of Y-chromosome scaffolds which were extensively validated by PCR.

**Conclusions:**

The high-quality genome generated represents a critical tool in olive fruit fly research. We provide an extensive RNA-seq data set, and genome annotation, critical towards gaining an insight into the biology of the olive fruit fly. In addition, elucidation of Y-chromosome sequences will advance our understanding of the Y-chromosome’s organization, function and evolution and is poised to provide avenues for sterile insect technique approaches.

## Background

Some animals have always been “more equal” than others.^1^ For many researchers, working on anything ranging from classical genetics to developmental biology to modern genomics, the “most equal” animal has been *Drosophila melanogaster.* Despite Drosophila’s insignificant agricultural or medical importance, it became, in 2000, the first complex eukaryote whose genome was sequenced and assembled [1]. More important insect genomes, like that of the malaria mosquito *Anopheles gambiae*, followed soon after [2]. However, non-model insects or insects with less important public health or global agricultural impact had a much harder time having their whole genomes sequenced. This held back several advances that would be based on understanding their genomes, including tools for developing alternative pest control methods. Gradually, advances in DNA sequencing technologies that dramatically reduced the cost and time to sequence an organism’s entire genome made sequencing of numerous insect genomes a reality. In 2011, the “i5k” initiative was launched to provide the genomic sequences of 5000 insect or related arthropod species [3]. In this project, the onus was placed on individual labs with a specific interest in these genomes to organize the sequencing, analysis, and curation of their genomes [4]. Eight years later the target is still far from being achieved. As of March 2019, only 1219 insect genomes had been registered in the National Center for Biotechnology Information (NCBI) and only 401 of them have had at least a draft genome assembly [5].

The goal of sequencing 5000 insect genomes was not put as a mere technological challenge. Sequencing information can enormously help the understanding of insect biology as well as provide insights for environmentally friendlier means of control. For example, accurate genome sequence information is now the basis for precise CRISPR-based genetic manipulation and genome editing (e.g., Kyrou et al. [6]), or for designing RNAi-based species-specific and eco-friendly insecticides (for a recent review see Vogel et al. [7]). Furthermore, the genomic diversity of ecotypes, geographical isolates and related species can be combined with genome-wide association studies (GWAS) and reveal the genetic components of certain traits and adaptations such as insecticide resistance [8, 9]), geographical polymorphism [10, 11]) or host adaptation [12]. Despite this importance, insect whole genome sequencing (WGS) projects are not advancing at the anticipated pace. Firstly, small physical insect sizes might not allow enough quantities of DNA to be isolated from a single individual. Secondly, high population polymorphism and/or difficulty to breed for genome homozygosity renders genome assembly efforts particularly difficult [13]. Therefore, it is critical to establish methodological approaches that will allow the de novo sequencing of insect genomes at high quality and low cost if the i5k target is to be achieved.

The ideal sequencing approach should provide very long reads (in order of megabases, Mb) with single base-pair resolution, very low error rate, and low cost. However, no such platform currently exists. Short-read sequencing technologies, such as ‘single nucleotide fluorescent base extension with reversible terminators [14]’ commonly referred to as Illumina sequencing (Illumina Inc.), deliver massive numbers of relatively cheap short (50–300 bp) high quality reads but de novo genome assemblies from such technologies are often fragmented. On the other hand, long-read sequencing technologies such as nanopore sequencing from Oxford Nanopore Technologies (ONT) and Single Molecule Real-Time (SMRT) sequencing from Pacific Biosciences Inc. (PacBio) which deliver long reads have relatively low throughput and high raw-read error rates. However, assemblies from these technologies are much more contiguous yielding completely closed genome assemblies for small organisms like prokaryotes [15]. To benefit from the pros of each sequencing technology, hybrid approaches that aim to sequence organisms using different approaches and then combine the data, either at the level of error correction of reads or scaffolding and gap-closing of assemblies, are increasingly widely applied (reviewed elsewhere [16]). Hybrid genome assemblies have shown more accuracy and contiguity [15, 17], and are now a preferred approach to de novo genome assembly.

The linked-reads technology [18, 19] from 10x Genomics (CA, USA) is a relatively new genomic library preparation approach. Conceptually, a single ultra-long DNA fragment is captured into an oil emulsion droplet (also called GEM or partition) and sampled along the length of the fragment using oligonucleotides bearing the same molecular barcode for each partition. Pooling and Illumina sequencing of all barcoded oligos and computationally linking all oligos taken from the same DNA molecule using the bespoke Supernova assembly tool [20] provides a new powerful approach for using short-read technologies in de novo genome assembly. This method has previously been applied to insect genomes with varying levels of success [21, 22]. This is probably partly because this entire methodology is optimized around human genomes and genomes of similar size, while for genomes of significantly smaller sizes, optimization of assembly parameters is needed [20].

In the current manuscript we present several technological advances that were developed in order to sequence the entire genome of a non-model organism but one of high agricultural significance, the olive fruit fly (*Bactrocera oleae*), whose genome size was initially estimated to be 322 Mb using qPCR [23]. The olive fruit fly belongs to the Tephritidae family of insects, a family that contains some of the most important agricultural pests world-wide, such as the Mediterranean fruit fly (medfly, *Ceratitis capitata*), the oriental fruit fly (*Bactrocera dorsalis*), the Mexican fruit fly (*Anastrepha ludens*), the Australian Queensland fruit fly (*Bactrocera tryoni*) and others. Olive fruit flies are the major pest of wild and commercially cultivated olives trees causing an estimated annual damage of USD 800 million [24, 25], since chemical insecticides do not fully protect a tree from being infested. Despite its economic importance in olive producing countries, several peculiarities of the olive fruit fly’s biology (e.g., difficulty in rearing, high natural homozygosity, lack of phenotypic mutations) made the development of classical genetics tools an impossible task. More recently, however, the olive fruit fly has been the subject of several molecular and transcriptomics studies [26–28] (Reviewed in Sagri et al. [29]).

Another particularity of the olive fruit fly is the fact that it possesses a very small Y chromosome [30, 31], karyotypically appearing as the ~ 4 Mb dot chromosome IV of *D. melanogaster* [32]. Among organisms that employ an X-Y chromosome system, as does the olive fruit fly, the Y chromosome has been notoriously difficult to assemble due to its heterochromatic and repetitive nature. For example, 80% of the *Drosophila melanogaster* Y chromosome is made up of repeats [33]. In most genome sequencing projects, the Y chromosome sequence is fragmented into many small, unmapped scaffolds [34]. Additionally, only a few genes reside on the Y chromosome and most of them are characterized by the presence of small exons, gigantic introns, and very little conservation among species even of the same family [35]. Therefore, Y chromosome assembly presents a unique challenge. In the olive fruit fly, the Y chromosome encompasses the male determining factor, *M*, that had remained elusive for over 30 years [36]. The *M* factor is the initial switch of the sex-determining cascade in tephritids, a switch that has been speculated to differ from the one used by the model dipteran Drosophila (for a review see [37]). The *M* factor has recently been identified in the medfly and a few other tephritids, including the olive fruit fly [38], but the details of the sex determination cascade remain unclear. Unraveling this cascade and identifying other genes that reside on the Y chromosome, probably involved in male fertility, will shed light on the evolution of a major developmental pathway in most animals, as well as the evolution of the sex chromosomes themselves [39, 40].

Here, we describe the whole genome sequence of the olive fruit fly, generated as a hybrid assembly using the 10x Genomics linked-reads assembly as the backbone followed by scaffolding and gap-filling with Illumina mate-pair reads, and long-reads from PacBio and ONT. This genome has a scaffold N50 of 4.69 Mb and L50 of 30 making it one of the most contiguous Tephritidae genomes in the current NCBI genome catalogue. We also identified Y chromosome-specific scaffolds and present the first assembly of the *B. oleae* Y chromosome that will be instrumental in the elucidation of the regulation of the *M* factor and the structure and evolution of the entire Y chromosome. We also provide 12 short-read RNA-seq datasets from different tissues and/or development stages which add extensive characterization of this organism.

## Results

**Fig. 1:**
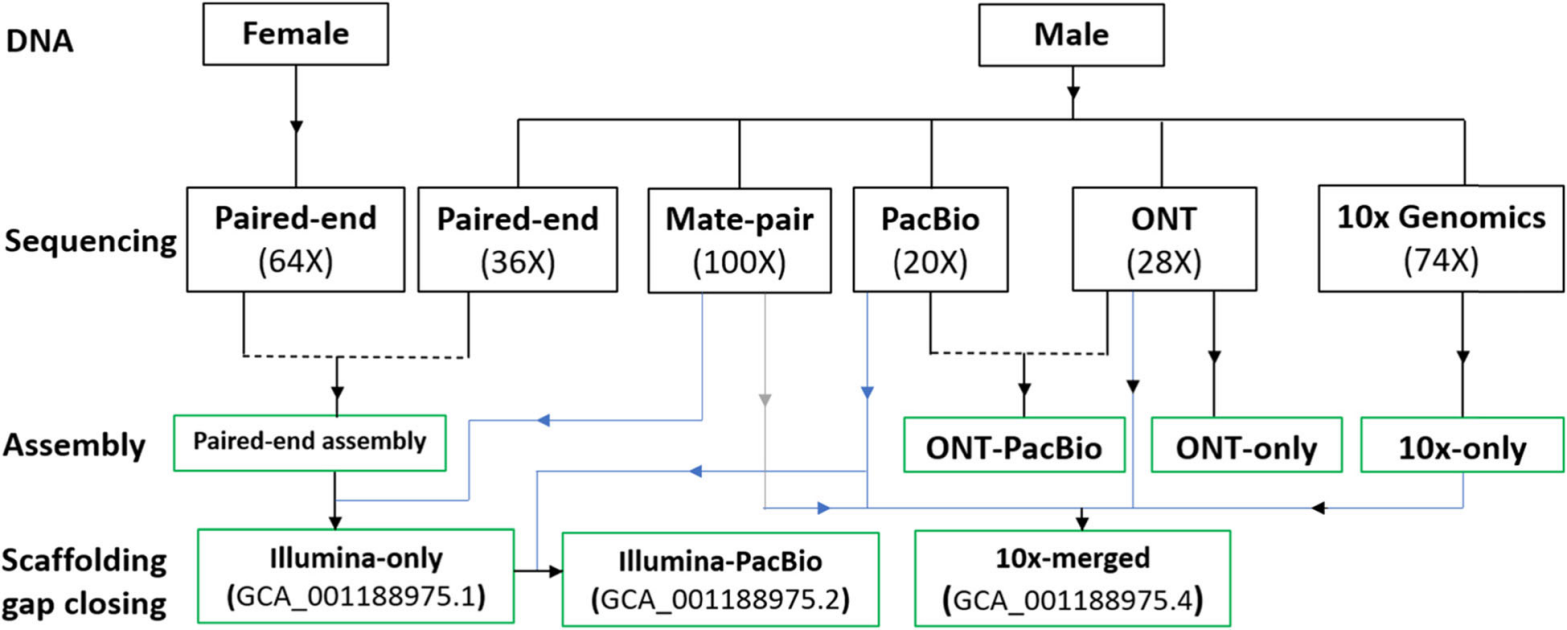
Schematic of the method used to generate the different assemblies. DNA extracted from adult female and/or male insects was used to generate sequencing libraries for; Illumina paired-end (PE, 64X and 6X coverage, respectively), mate-pair (MP, 100X coverage), 10x Genomics linked-reads (100X coverage generated but 74X was found optimal for genome assembly), Pacific Biosciences (PacBio, 20X coverage), and Oxford Nanopore Technologies (ONT, 28X coverage). Independently generated assemblies are shown, and assemblies generated from scaffolding and gap scaffolding are shown with their GenBank accession numbers. Arrows indicate the final resulting assemblies while arrow heads indicate the samples or datasets used to generate the final assemblies

In order to generate a high-quality genome assembly of the olive fruit fly we undertook a multistep process that consisted of different sequencing and assembly approaches (Fig. 1, Supplementary Figure S1). First, we generated sequence data using short-read and long-read sequencing platforms that was used to generate a hybrid assembly (GenBank accession GCA_001188975.2). We then used the 10x Genomics linked-reads technology to generate an independent haplotype-resolved assembly. The final steps involved scaffolding and gap-closing of the 10x assembly using mate-pair and long-reads and then finally polishing to generate the final assembly (GenBank accession GCA_001188975.4). The steps undertaken and the resulting assemblies are detailed below.

### Genome assembly using Illumina paired-end, mate-pair and PacBio reads

Our initial assembly was performed using two Illumina short insert paired-end (PE) libraries made separately from male and female flies, the sequencing of which yielded 36X and 61X theoretical coverage, respectively (see Supplementary Table S1). Male and female reads were assembled together using a short-read assembler, Ray [41], with a kmer of k41 which produced the largest scaffold. The assembly was further scaffolded with 100X coverage from three mate-pair (MP) libraries using SSPACE [42], and then gap-filled using 20X coverage of reads generated with SMRT technology from Pacific Biosciences (PacBio). This resulted in a final assembly that was submitted to NCBI (GenBank assembly accession: GCA_001188975.2). The submitted assembly had a total length of 471,780,370 bases with a scaffold N50 length of 139,566 bp reached with 474 scaffolds (Table 1, Supplementary Table S2). GCA_001188975.2 was also submitted to i5k [3].

**Table 1:** Statistics for the main *B. oleae* genome assemblies generated

**GenBank Accession**	**Name**	**# scaffolds/contigs**	**Total length (Mb)**	**Largest contig (Mb)**	**N50 (Mb)**	**L50**	**# N’s per 100 kb**
**GCA_001188975.2**	Illumina-PacBio	36,198	472	5.1	0.14	474	10,853.91
**GCA_001188975.4**	10x-All	39,141	489	19.4	4.69	30	5493.82

Quality metrics were generated using Quast [43]. N50 value is the scaffold/contig length at which half of the genome is contained in scaffolds/contigs at or above that length. L50 is the number of contigs needed to reach N50

### Utilization of linked-reads to generate a *Bactrocera oleae* assembly

**Fig. 2:**
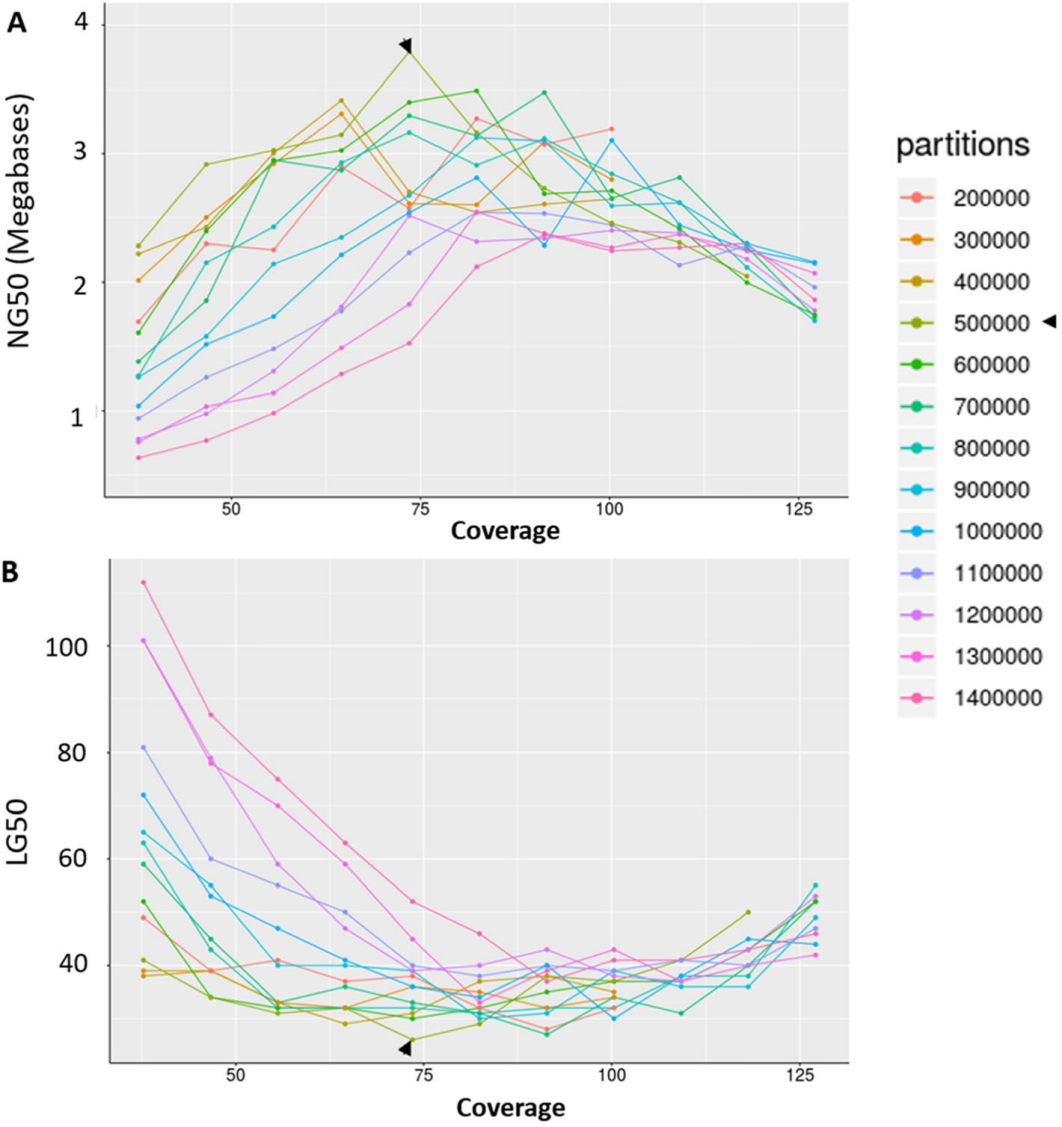
Optimization of number of partitions and coverage for the Supernova assembler. Different number of partitions were randomly selected using the partition (GEM) barcodes while also varying the number of reads per partition to optimize the coverage. These were provided as input for the assembler. For each resulting assembly the NG50 length and LG50 count were calculated with genome size assumed to be 320 Mb [23]. NG50 value is the scaffold/contig length at which half of the genome (~ 160 Mb) is contained in scaffolds/contigs at or above that length. LG50 is the number of contigs needed to reach N50. Arrow heads indicate optimized parameters

The 10x Genomics platform which generates linked-reads has great potential to yield high quality assemblies in terms of base accuracy, contiguity, and phasing. High molecular weight DNA was extracted from male ‘Demokritos’ strain of the olive fruit fly which has been a lab strain for over 45 years. This strain has been maintained in our lab for over 15 years with no addition of wild flies. Unlike the *C. capitata* genome [44] that required inbreeding of the ISPRA strain for 20 generations which resulted in low heterozygosity (0.391%), the Demokritos olive fruit fly strain used in the current research was already of low heterozygosity (0.401%, Supplementary Figure S2). This is due to the huge bottleneck that the olive fruit fly undergoes during domestication [45], the large number of years that the Demokritos strain has spent in laboratory conditions (> 45) and, probably, other reasons that have to do with the biology of the insect (e.g., strict monophagy of the larva). Linked-reads library preparation (done at 10x Genomics, San Francisco, CA, USA) and sequencing resulted in 100X coverage worth of data which was assembled using the bespoke Supernova assembler. Because genome assembly with 10x Genomics data was only optimized for human genomes [20], we derived our optimized parameters. Specifically, we performed several rounds of genome assemblies varying the coverage depth and number of partitions and compared the resulting NG50. The assembly NG50 increased with increasing coverage up to a peak above which the assembly NG50 dropped for all partitions tested (Fig. 2a). Increasing coverage had the opposite effect on genome LG50 (Fig. 2b). The best assembly was obtained with 74X coverage and 500,000 partitions which corresponded to 331 reads per partition. The optimized parameters (number of partitions to use, reads per partition, and coverage) were used to generate an assembly of 434.81 Mb with a scaffold N50 of 2.16 Mb, with the largest scaffold stretching 12 Mb. The L50 was only 44 (Supplementary Table S3, Supplementary Figure S3). This assembly is here referred to as 10x-only. Using this assembly as the backbone, several scaffoldings were performed to increase genome contiguity.

### Scaffolding and gap-closing of the linked-reads assembly

We explored the effectiveness of combining the 10x-only assembly with short-reads and long-reads. Oxford Nanopore technologies (ONT) and Pacific Biosciences currently generate the longest raw reads of any commercially available DNA sequencers with ONT having no theoretical limits [46]. This provides potential to significantly increase assembly contiguity. High molecular weight DNA was extracted from a pool of adult male flies and used to prepare ONT and PacBio sequencing libraries, the sequencing of which resulted in a theoretical coverage of 28X and 20X, respectively. The ONT reads had an N50 of 11 kb with the longest read generated being 780 kb. The short and long reads enabled scaffolding and gap-closing of the 10x-only assembly (see Supplementary Table S3 and Supplementary Figure S3 for a summary of the results). Using SSPACE, mate-pair sequences were used to scaffold the 10x-only assembly. This had a noticeable improvement on the 10x-only assembly increasing the N50 from 2.16 to 3.26 Mb (51% increase) at the expense of including gaps between scaffolded contigs (gaps increased from 3543.94 to 10,744.3 N’s per 100 kb). Scaffolding the 10x-only assembly with PacBio reads (20X coverage) using PBJelly increased the 10x-only scaffold N50 from 2.16 to 3.77 Mb (74% increase) and reduced the L50 to 32 scaffolds. Scaffolding the 10x-only assembly using ONT reads (28X coverage) had the biggest improvement on contiguity. The scaffold N50 more than doubled from 2.16 to 4.59 Mb (112% increase) and the L50 was reduced from 44 to 29 scaffolds. Further, the ONT reads increased the largest scaffold from 12 Mb to 19.3 Mb. Scaffolding with either PacBio or ONT had similar effects on assembly gaps (reducing from 3544 to 3538 and 3532 N’s per 100 kb, respectively).

The final assembly was generated by combining all technologies. Scaffolding the 10x-only assembly first with mate-pairs then PacBio followed by ONT produced the highest contiguity. The final assembly was polished using Pilon and submitted to NCBI with assembly name “MU_Boleae_v2” (GenBank accession; GCA_001188975.4). This is the most contiguous *B. oleae* genome assembly to date (see Supplementary Figure S4 for comparison to the previous assembly). The total assembly size is 488.86 Mb, with scaffold N50 of 4.69 Mb, 36,198 total scaffolds, and scaffold L50 of 30 (Table 1). This genome size is slightly larger than the 446 Mb predicted using kmer analysis [47] and significantly larger than 322 Mb predicted by qPCR [23]. This genome size is similar to other closely related species (*Ceratitis capitata*, 479 Mb [44]; *Bactrocera dorsalis*, 414 Mb; *Zeugodacus cucurbitae*, 374 Mb). Generally, insect genome sizes differ greatly from 68.5 Mb (Midge, *Clunio tsushimensis*) to 16.5 Gb (Mountain grasshopper, *Podisma pedestris*), with median of 498.8 Mb [48]. Dipteran insects, however, have smaller genomes ranging from 68.5 Mb (Midge, *Clunio tsushimensis*) to 1.8 Gb (Mosquito, *Aedes zoosophus*), median 224.9 Mb [48]. The olive fruit fly genome at 485 Mb is about the median insect size and about twice the median Dipteran genome size.

### Identification of sex chromosome sequences and Y chromosome assembly

**Fig. 3:**
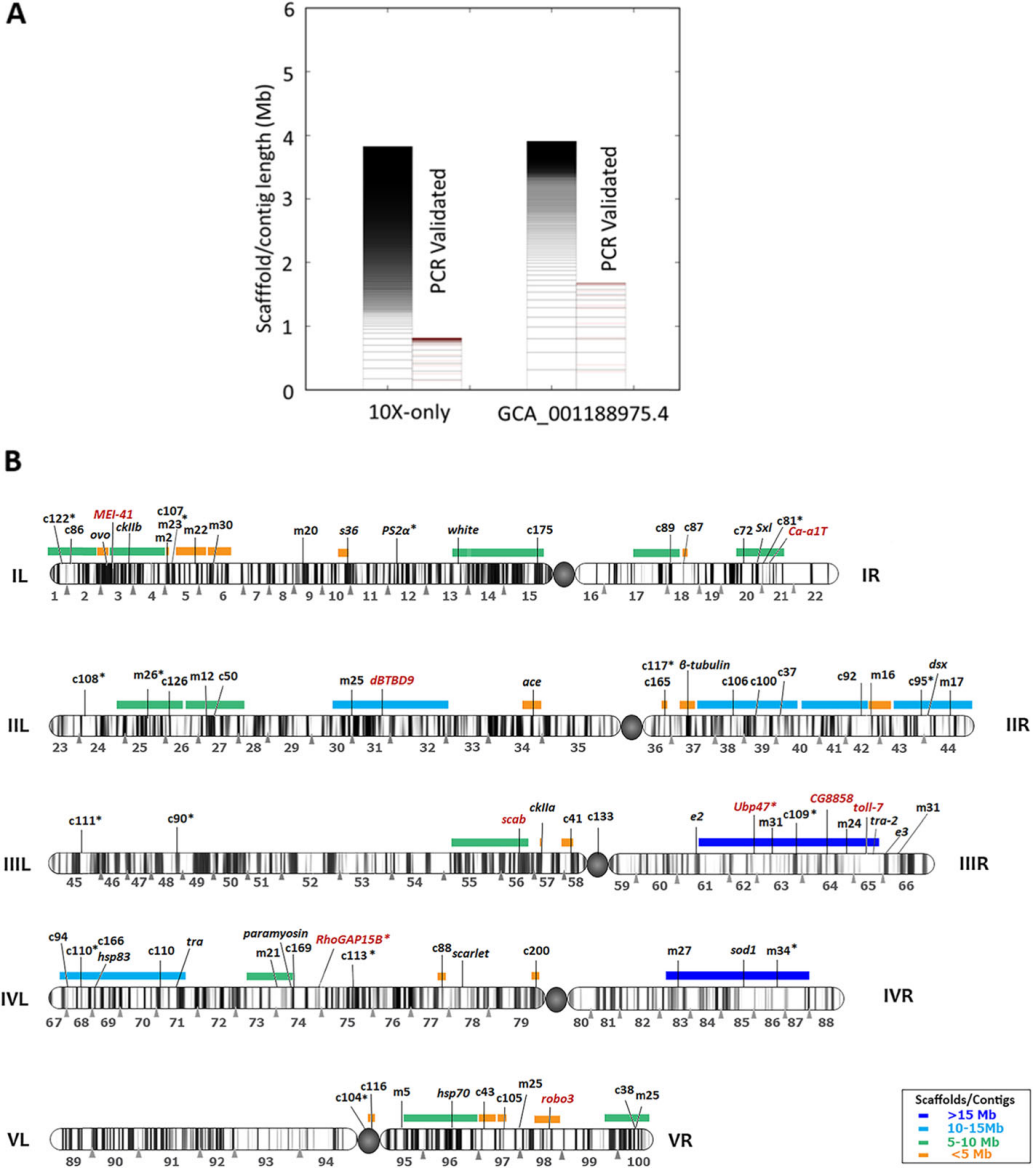
*B. oleae* polytene chromosomes mapping of molecular markers Y chromosome assembly. **a** Plot showing Y chromosome scaffolds/contigs identified in 2 different assemblies (Supplementary Table S4). The Chromosome Quotient (CQ) method [49] was used to identify Y chromosome scaffolds. The scaffolds/contigs are ordered from longest at the bottom to shortest at the top. For each assembly the total scaffolds/contigs are shown in left bars while the PCR validated scaffolds/contigs are the right bars. The approximate location of the PCR primer on the scaffold/contig is shown in pink. **b** Schematic representation of *B. oleae* polytene chromosomes including all mapped markers (tags) and the scaffolds assigned to chromosomes. Previously and currently mapped markers are indicated with black and red letters, respectively, above chromosomes. Colored horizontal bars above chromosomes indicate scaffolds/contigs in the GCA_001188975.4 assembly that were localized to chromosomes using mapped markers. More than one tags on a specific scaffold is informative of its physical orientation. m## corresponds to microsatellite markers number ##; c## corresponds to EST marker number ## [26, 50]; newly mapped genes in the current study are presented in full names or abbreviations (Supplementary Table S6); “*” indicates the tags that were not found on the anchored contig or gave ambiguous alignment results. The centromere is shown as a filled circle. (see Supplementary Table S7 for detailed information)

In order to find putative X or Y chromosome scaffolds we used the Chromosome Quotient (CQ) method [49]. The CQ reflects the median ratio of female to male reads coverage when these reads are separately aligned to a male genome assembly. The CQ values will cluster around zero, one, or two for Y, autosome, and X scaffolds, respectively. Using the repeat masked version of the final assembly (GCA_001188975.4), which was generated from male olive fruit fly DNA, male and female short Illumina reads (40X coverage of each) were independently mapped. Considering only the scaffolds with a CQ of 0, we obtained a total length of putative Y-chromosome of 3.9 Mb with 873 scaffolds (Fig. 3a). We similarly determined putative Y-chromosome scaffolds from other assemblies and compared them (Supplementary Figure S5, Supplementary Table S4). The GCA_001188975.4 Y scaffolds showed high contiguity with a scaffold N50 of 60 kb and the largest scaffold being 318 kb. The size of our assembled *B. oleae* Y chromosome at 3.9 Mb is very similar to the predicted size of 4 Mb [32] and thus likely captures most of it. The X chromosome scaffolds identified in the GCA_001188975.4 assembly totaled 6 Mb.

### Validation of Y-chromosome specific scaffolds

To validate the Y scaffolds identified using the CQ method, 85 primer pairs (see Supplementary Table S5), chosen from the largest scaffolds, were designed to amplify regions of the different Y-linked scaffolds by polymerase chain reaction (PCR) using either male or female genomic DNA as template. When a primer pair resulted in the amplification of the expected size band with male genomic DNA only, we concluded that its corresponding scaffold was Y-specific. However, it was expected that some primers might represent homologous regions between X and Y chromosomes and thus have a product both in male and female samples, albeit at a lower level in the females. Partial homology with autosomal sequences was also expected. Quantitative real time PCR (qPCR) offers a much more precise method to detect such differences. Therefore, lower male qPCR cycle-threshold (Ct) amplification values than female should indicate that the respective primer pair corresponded to a Y-specific scaffold. Nine primer pairs gave no amplification, 11 gave ambiguous results and require further examination, while 30 equally amplified male and female gDNA. A total of 1.7 Mb out of 3.9 Mb in GCA_001188975.4 assembly was thus confirmed as Y-chromosome (Supplementary Figure S6). To further validate scaffolds potentially containing Y chromosome sequence, we used the Y chromosome Genome Scan method (YGS, [51]) which retrieved 1196 scaffolds totaling 3.9 Mb. Of these scaffolds, 271 scaffolds totaling 2.7 Mb or 68% of putative Y chromosome had also been identified using the CQ method. Further, the scaffolds identified using YGS method contained all the PCR confirmed scaffolds. The Y chromosome, however, remains difficult to assemble with orthogonal methods yielding slightly differing results.

### Generation of chromosome markers and scaffolds assignment to chromosomes

The olive fruit fly has well-characterized cytogenetic maps derived from polytene chromosomes [52], which enables the determination of the exact position of scaffolds containing specific markers. Further, scaffolds containing more than one mapped marker can be oriented on the chromosomes. We, therefore, used already mapped and newly generated molecular markers in order to position sequenced scaffolds on the chromosomes. The markers used included 35 expressed sequence tags (ESTs) [26], 16 microsatellites [53], and 19 previously localized heterologous genes [50, 52, 54] providing 70 tags in total. As part of the current work we generated 9 new markers (Supplementary Table S6) and mapped their position on salivary gland polytene chromosomes by in situ hybridization (Fig. 3b). All 79 tags were aligned using Minimap2 [55] in splice-aware mode and also BLAST’ed. against the GCA_001188975.4 genome in order to assign scaffolds/contigs to chromosomal arms. However, 25 tags gave ambiguous alignment results and were not used further. The remaining 54 tags allowed the physical mapping of 36 contigs with a total length of 200 Mb, corresponding to 41% of the total genome size (Fig. 3b, Supplementary Table S7). Among the 36, 10 scaffolds totaling 106 Mb contained more than 1 marker and could thus be oriented. Addition of the X and Y chromosome scaffolds totaling 6 and 4 Mb, respectively, that were identified using the CQ method brought the total percentage of the genome assigned to chromosomes to 43% (Fig. 3b, Supplementary Figure S7).

### Evaluation of assembly completeness

We evaluated genome completeness using 3 metrices; genome size, alignment of RNA-seq data, and recovery of basic universal single copy orthologs (BUSCOs [56]). The frequence of k-mers of length = 23 bases was calculated using BBMAP [57] followed by genome size estimation using GenomeScope [58] (Supplementary Figure S2). The *B. oleae* genome size was estimated at 439.8 Mb. This was close to the final genome assembly size of 489 Mb. We generated RNA-seq data from 12 different tissues/stages and aligned them to the GCA_001188975.4 assembly. RNA-seq data alignment rates ranged from 85 to 96% (Supplementary Figure S8), which is similar to the expected ranges of 70 and 90% [59]. Perhaps owing to the low heterozygosity, the diploid genome had similar alignment rates to the GCA_001188975.4 assembly which is a single haplotype (Supplementary Figure S8). Nonetheless, we separately provide the second haplotype (SRA index SRR9678778). BUSCOs analysis showed that across the 4 lineages analysed; Eukaryota, Arthropoda, Insecta, and Diptera, 99.3, 99.2, 99.2, and 98.1% of the genes surveyed were captured in the GCA_001188975.4 assembly (Supplementary Figure S9). The complete Diptera BUSCOs recovered in the GCA_001188975.4 assembly (98.1%) were higher than the previous assembly GCA_001188975.2 (95.6%) showing an improvement in assembly quality.

### Identification of symbiont derived sequences

Sequences that belong to bacterial contaminants or symbionts in the GCA_001188975.4 assembly were identified using a similar approach applied to the Mediterranean fruit fly [44]. We identified small fragments that displayed homology with *Wolbachia* sequences. The biggest fragment identified was 831 bp in length exhibiting a similarity of 89.2% with an ankyrin from the *w*Mau *Wolbachia* strain. In total 14 fragments were identified with a size range from 259 to 855 bp. No *Cardinium* and *Spiroplasma* sequences were present either in the raw dataset or in the assembled contigs.

Our second approach using bacterial complete and draft genomes deposited in NCBI (assessed June 2019) revealed the presence of sequences affiliated mainly with *Agrobacterium rhizogenes*, *Deftlia* sp., and *Agrobacterium tumefaciens* which were found to be present in 17.5, 15.9 and 8% of all scaffolds, respectively. Most of the sequences identified (84.6%) had a length of 100 to 2500 bp. Eight alignments were spanning more than 20,000 bp. Alignments smaller than 100 bases were considered as noise and were not included in the analysis. The percentage of sequence similarity was between 100 and 65% with 43.7% exhibiting a similarity between 90 and 100%. It’s worth noting that no sequences of the olive fruit fly symbiont *Candidatus* Erwinia dacicola were identified which confirms previous reports that this symbiont was lost upon the laboratory domestication and the artificial rearing of this insect pest species [60]. Nevertheless, trimming or removal of scaffolds with evidence of bacterial DNA was guided by NCBI assembly quality check. NCBI quality control and contamination check identified 134 scaffolds/contigs totaling 147 kb with bacterial origin which were removed. A further 981 scaffolds/contigs totaling 3.92 Mb were suppressed due to possession of bacterial gene models.

### Transposable element identification and annotation

Discovered in the late 1940s in maize [61], transposable elements (TEs) have since been found in almost all eukaryotic organisms surveyed except for *Plasmodium falciparum* [62]*.* The highest TE subdivision, Class, comprises 2 groups; Class I and Class II. Class I comprises retrotransposons which utilize a ‘copy-and-paste’ mechanism of transposition with an RNA intermediate while Class II comprises DNA transposons that utilize a ‘cut-and-paste’ transposition mechanism with a DNA intermediate. The major orders in Class I are; LTR, DIRS, PLE, LINE, and SINE. Major orders in Class II are; TIR, Crypton, Helitron, and Maverick. TEs are further subdivided down to subfamily level. Virtually all these types of TEs are found in insect genomes with Class I elements being more predominant [63]. LTR for example are the most predominant in *D. melanogaster*, followed by LINEs, and TIR [64, 65]. In insects, TEs play a role in mutagenesis, inter and intra-chromosomal rearrangements, evolution of sex chromosomes, and genomic adaptation (reviewed in [60]). Discovery methods of TEs can be divided into 2; those that rely on raw sequence reads and those that rely on an assembled genome [66]. Due to the challenges in detecting and annotating TEs, combining tools has been shown to improve detection [67, 68]. We used the PiRATE TE detection pipeline [67] (Supplementary Figure S10), which includes 9 genome based TE identification tools, to derive a TE library. The TE library was classified using PASTEC [69] and then used to annotate and mask the genome using TEannot [70] and RepeatMasker, respectively.

PASTEC classification of the repeat library (Table 2) showed that Class II TEs were most numerous of all repeat elements (45%). This contrasts with *C. capitata* where Class I are the most numerous (55.9% of TEs) [44]. Terminal inverted repeat (TIR) transposons subclass, which includes the *Tc1-mariner* superfamily, was most numerous accounting for 29% of all TEs. However, the *B. oleae* and *C. capitata* percentages of LTR elements (15.9% vs 15.7%, of all TEs respectively) and DNA transposons (45.15% vs 44.1%, of all TEs respectively) are similar [44]. *C. capitata* genome was also assembled using long-reads and thus, repeated regions should be fairly well captured.

**Table 2:** Classification of transposable elements (TE) identified in *B. oleae* genome

**Class**	**Order**	**Number**	**Percentage**
Class I	DIRS	30	0.06
	LARD	1111	2.12
	LINE	7802	14.85
	LTR	8339	15.88
	PLE	5	0.01
	SINE	206	0.39
	TRIM	831	1.58
	No order	78	0.15
	Several orders	11	0.02
	**Total**	**18,413**	**34.99**
Class II	Helitron	7700	14.66
	MITE	113	0.21
	Maverick	133	0.25
	TIR	15,301	29.13
	No order	481	0.92
	Several orders	21	0.04
	**Total**	**23,749**	**45.13**
Simple Sequence Repeats		100	0.19
No category		10,361	19.73
**Total**		**52,623**	**100**

Nine de novo and similarity based TE identification tools included in the PiRATE pipeline [67] were used to generate a library of TE followed by classification using PASTEC [69]

Genome repeat masking using the derived TE library and RepeatMasker showed that TE account for 34.94% of the *B. oleae* genome. In *Drosophila*, TE genome coverage is variable, ranging from 2.7% in *D. simulans* to 24.9% in *D. ananassae* [71] and is highly correlated with genome size [72]. In the more closely related species, *C. capitata*, TE constitute 18% of the genome [44]. In terms of genome coverage, Class II DNA transposons accounted for 16.15% of the genome while Class I retrotransposons accounted for 10% of the genome. We attempted to annotate the *B. oleae* TE down to superfamily level using TEannot (Supplementary Table S8) but only 5% of the genome was annotated. Nevertheless, among the annotated families, *Tc1-mariner* were the most numerous with 1.8 million copies. The *Tc1-mariner* are ubiquitous Class II TE that form the largest group of eukaryotic TEs [73]. In insects the *Tc1-mariner* superfamily shows the highest level of horizontal transfer [74]. Class II TE and particularly *Tc1-mariner* and *PiggyBac* TE are of huge significance in Tephritidae sterile insect technique (SIT) as they have been used in medfly control and could be useful in *B. oleae* control [75, 76].

### Functional genome annotation and curation

We performed extensive RNA sequencing of the olive fruit fly. RNA was extracted from 12 tissues and/or stages; 6 from female, 1 from male and 5 of mixed origin. The tissues and/or organs included eggs, larvae, pupae, heads, testes among others (Supplementary Table S9). RNA-seq data was collected from these tissues and stages since they were used to address other important questions of the *B. oleae* biology, such as the reproductive and the olfactory system [28, 29]. Between 29 and 55 million reads per sample were generated and used to perform de novo transcript assembly using Trinity [77]. This produced 133,003 transcripts with a median transcript length of 503 bp (Supplementary Table S10 and Supplementary Figure S11). The completeness of the assembly was evaluated by querying Arthropoda, Insecta, and Diptera Basic Universal Single Copy Orthologs (BUSCOs) in the assembly of which 99, 98.4, and 94.8% are present as complete (Supplementary Table S11) suggesting that the transcriptome captured most genes. Overall alignment rates of RNA-seq data ranged from 88 to 94% (Supplementary Figure S8).

A more comprehensive protein coding gene-prediction pipeline, JAMg [78], was used to derive a more complete transcriptome of the olive fruit fly, integrating the RNA-seq datasets as a source of evidence. This pipeline has previously been used to annotate other Tephritidae genomes with good comparison to NCBI eukaryotic annotation pipeline [44]. The JAMg derived official gene set (OGS) contains a total of 16,455 protein-coding genes. Further, 3920 genes (23.8%) are predicted to have variants (isoforms) giving a total of 25,885 isoforms. Excluding isoforms, the mean gene (exons and introns) and transcript (coding and non-coding exons only) length is 11,545 bp and 2109 bp, respectively, with the longest gene found to be 299,321 bp and the longest transcript being 61,439 bp. The top BLAST hit for the longest gene was *fruitless* which encompasses 131 kb genomic region in *D. melanogaster* [79] while the longest transcript was the 8 kb *D. melanogaster beta-spec*.

To determine the completeness of the JAMg transcriptome, Diptera BUSCOs were searched. Of the 2799 BUSCOs 2703 (96.57%) were captured. This is comparable to 99.3 and 99.4% identified in *D. melanogaster* and *C. capitata*, respectively (see Supplementary Figure S12 for comparison to 18 other insect proteomes). Alignment of RNA-seq data derived from 12 different tissues showed alignment rates from 64 to 77% (Supplementary Figure S8). Further, 55% of all predicted genes could be assigned to chromosomes while 45% were located on scaffolds/contigs that are not yet assigned to individual chromosomes (Supplementary Figure S13). Each *B. oleae* protein (or the longest protein for multi-isoform genes) was BLAST-searched against the Swiss-Prot database (Evalue of 0.0004). Out of the 16,455 genes, 10,505 (64%) had significant hits. Blast2GO [80] was used to retrieve domain and motif signatures via Interproscan [81] analysis followed by identification of gene ontology (GO) terms via mapping and assignment of GO terms to sequences through functional annotation. Except for 51, all proteins with BLAST hits could be mapped and annotated. The top GO terms in each of Biological function, Cellular function, and Molecular function categories are shown in Supplementary Figure S14.

The *B. oleae* mitochondrial genome (GenBank accession NC_005333.1) has been previously described [82] This 15.8 kb genome encodes 13 protein coding genes/subunits (NADH dehydrogenase, cytochrome b and c, ATP synthase), 22 tRNA genes and 2 rRNA genes (12S and 16S).

### Orthology and phylogeny relationship to other insects

**Fig. 4:**
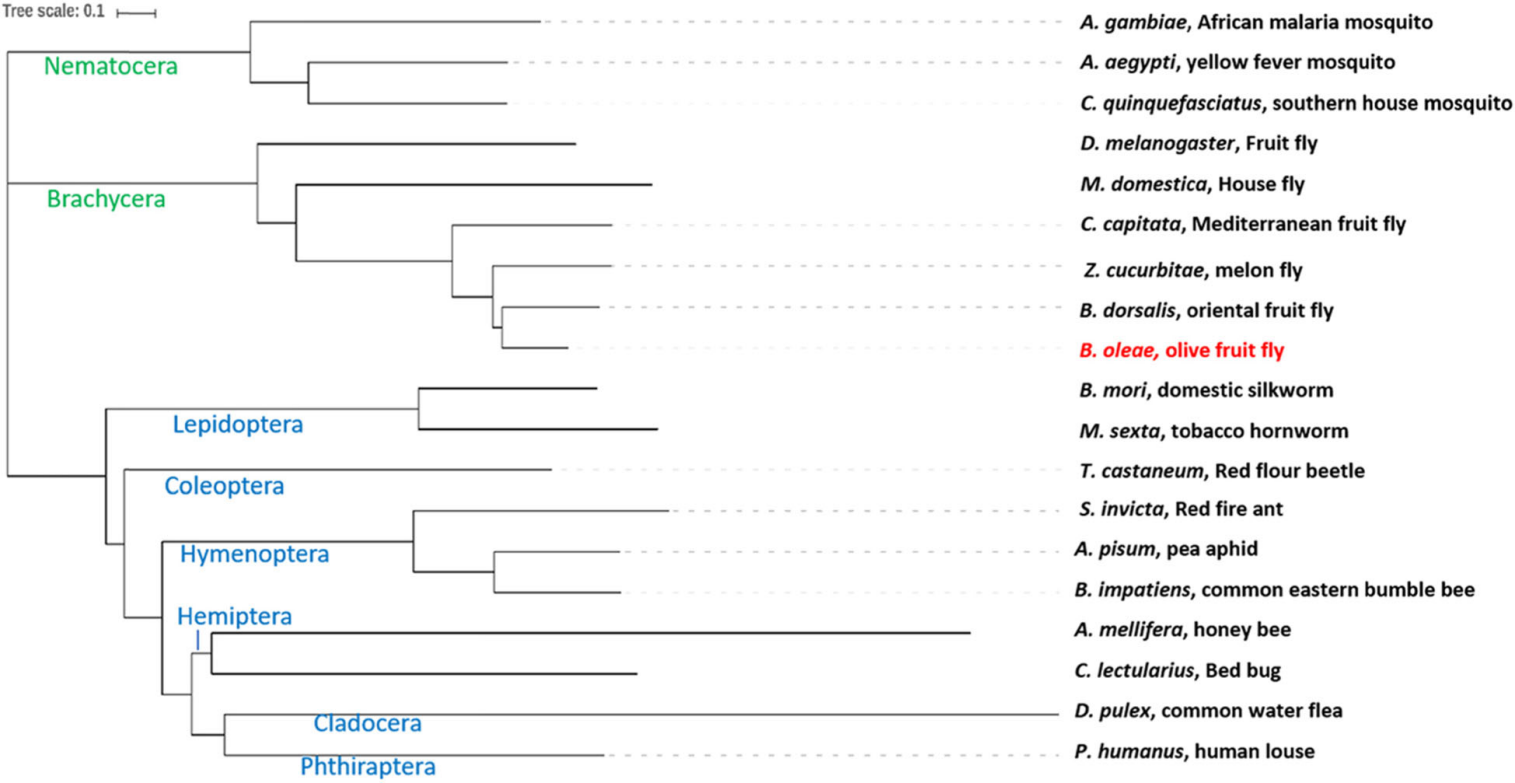
Phylogenetic relationship of *Bactrocera oleae* (olive fruit fly) and 18 other arthropods. Whole proteomes were used to infer pairwise distances of the 19 species using Prot-SpaM [84]. A phylogenetic tree was generated using Neighbor-Joining algorithm [84] implemented in T-REX [85] and viewed using iTOL [86]. See Supplementary Table S16 for sources of the proteomes used

Using complete proteomes, we analyzed phylogeny relationships between *B. oleae* and 18 other insects, 15 of which were previously analyzed but the authors used selected orthologs [44]. Traditionally, evolutionary relationships are inferred from multiple sequence alignment of selected homologous proteins. However, alignment-free methods which make use of whole proteomes rather than selected proteins have been shown to perform comparably [83]. We used Prot-SpaM [83] to infer pairwise distances of the 19 species. A phylogenetic tree (Fig. 4) was estimated using *Neighbor-Joining* algorithm [84] implemented in T-REX [85] and viewed using iTOL [86]. This un-rooted phenetic tree largely recapitulates the previously reported evolutionary tree [44] showing that *B. oleae* is more closely related to the other tephritid *Bactrocera dorsalis*, *Zeugodacus cucurbitae*, and *C. capitata* and more distantly related to *D. melanogaster*. The other insects were also well clustered according to their order or suborder.

**Fig. 5:**
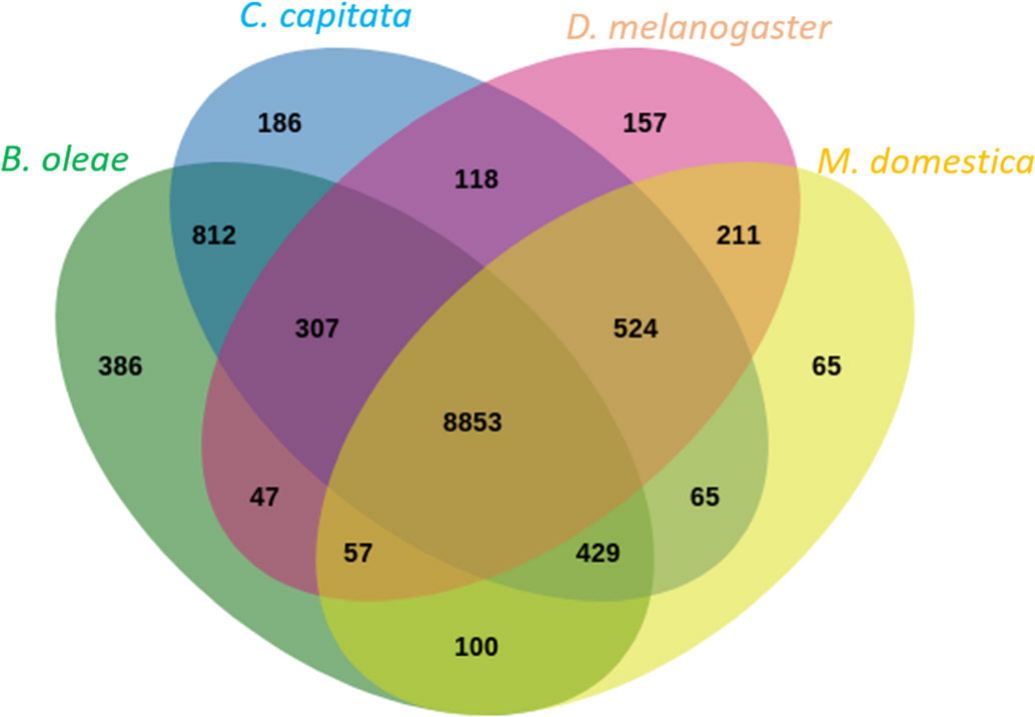
Venn diagram of shared orthogroups among *B. oleae, C. capitata, D. melanogaster, and M. domestica.* Orthologous proteins were identified and grouped using OrthoFinder [87]. Shared and unique orthogroups are plotted using Jvenn [88]. See Supplementary Table S16 for sources of the proteomes used

Orthologs among the 6 most closely related insects; *D. melanogaster, M. domestica, C. capitata, Z. cucurbitae, B. dorsalis*, and *B. oleae* were identified using OrthoFinder [87]. A total of 12,413 orthogroups were generated (Table 3, Supplementary Table S12). Out of a total of 144,022 total protein sequences 90% were assigned to an orthogroup. *D. melanogaster* and *B. oleae* had the highest number of proteins not assigned to an orthogroup; 16.6 and 16%, respectively. A total of 1395 orthogroups were identified that contain a single protein from each of the 6 species and another 7286 orthogroups that had one or more` protein from each species. As it would be expected, *B. oleae* shared more orthogroups with *C. capitata* than with *D. melanogaster* or *M. domestica* (Fig. 5, see Supplementary Figure S15 for a comparison of all 6 species).

**Table 3:** Summary of orthologous proteins among six most closely related Dipteran insects

	*B. dorsalis*	*B. oleae*	*C. capitata*	*D. melanogaster*	*M. domestica*	*Z. cucurbitae*
Total proteins	20,833	25,885	22,949	30,588	19,552	24,215
Proteins in orthogroups	20,296	21,745	22,135	25,523	16,931	23,477
Unassigned proteins	537	4140	814	5065	2621	738
Proteins in orthogroups (%)	97.4	84	96.5	83.4	86.6	97
Unassigned proteins (%)	2.3	16	3.5	16.6	13.4	3
Orthogroups containing species	11,417	10,991	11,294	10,274	10,304	11,521
Species-specific orthogroups	2	17	12	113	30	5
Proteins in species-specific orthogroups (%)	6	90	71	490	131	23
Proteins in species-specific orthogroups (%)	0	0.3	0.3	1.6	0.7	0.1

*Bactrocera dorsalis, Bactrocera oleae, Ceratitis capitata, Drosophila melanogaster, Musca domestica,* and *Zeugodacus cucurbitae.* Orthologous proteins were identified and grouped using OrthoFinder [87]. % = percentage, See Supplementary Table S16 for the source of proteins used

### Identification of developmental stage-specific genes

**Fig. 6:**
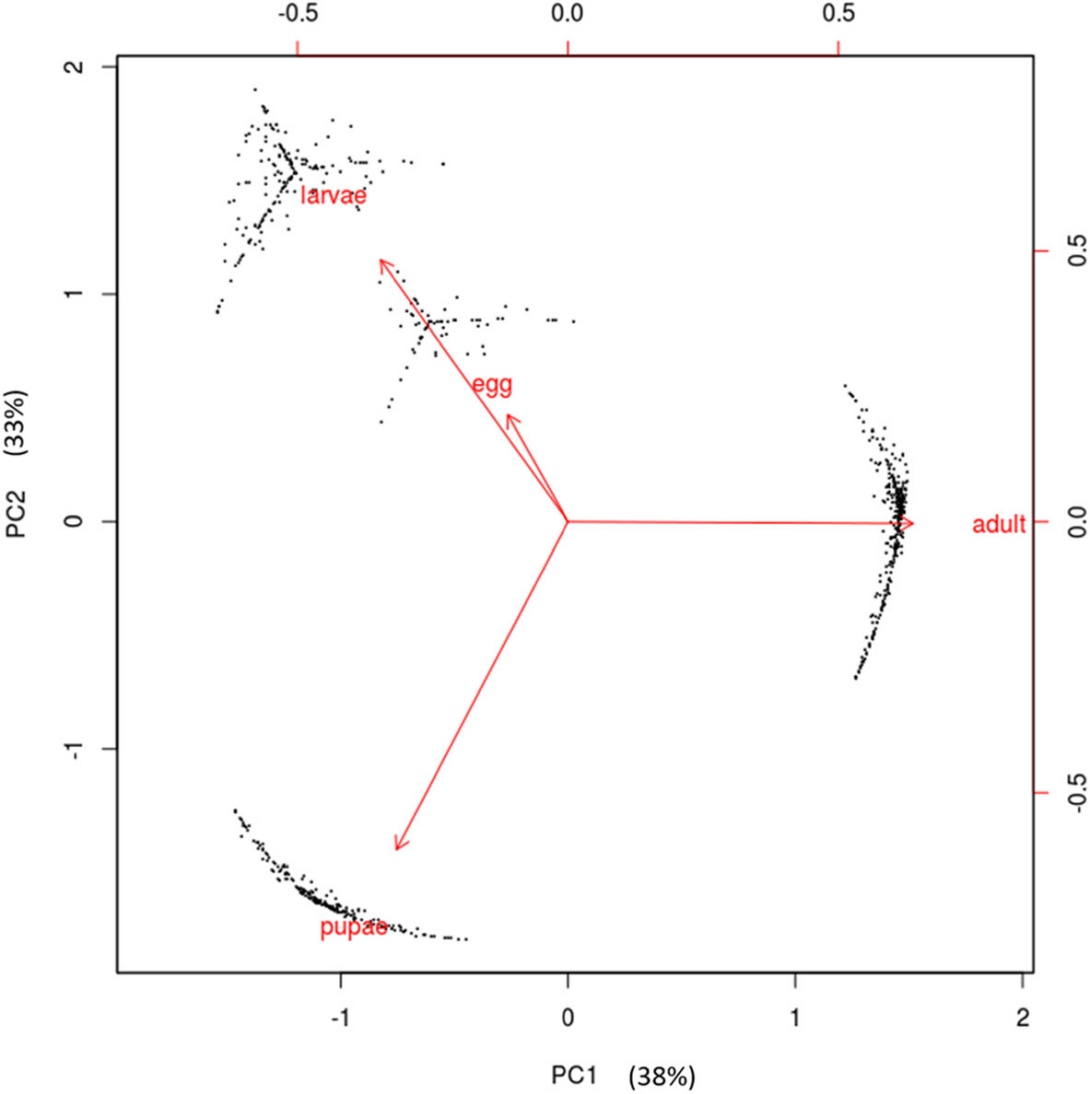
Principle component analysis (PCA) of 1100 most variable genes among the 4 metamorphotic stages. Gene expression (transcripts per million) was calculated for each of the stages; egg, larvae, pupae, and adult using RSEM [94]. A coefficient of variation was determined for each gene and used to determine the most variable genes. Eigenvector coordinates for the stages (egg, larvae, pupae, and adult) on the first 2 components are shown in red. Coordinates of the individual genes on the first 2 principle components (circle of correlation) are shown as black dots

**Fig. 7:**
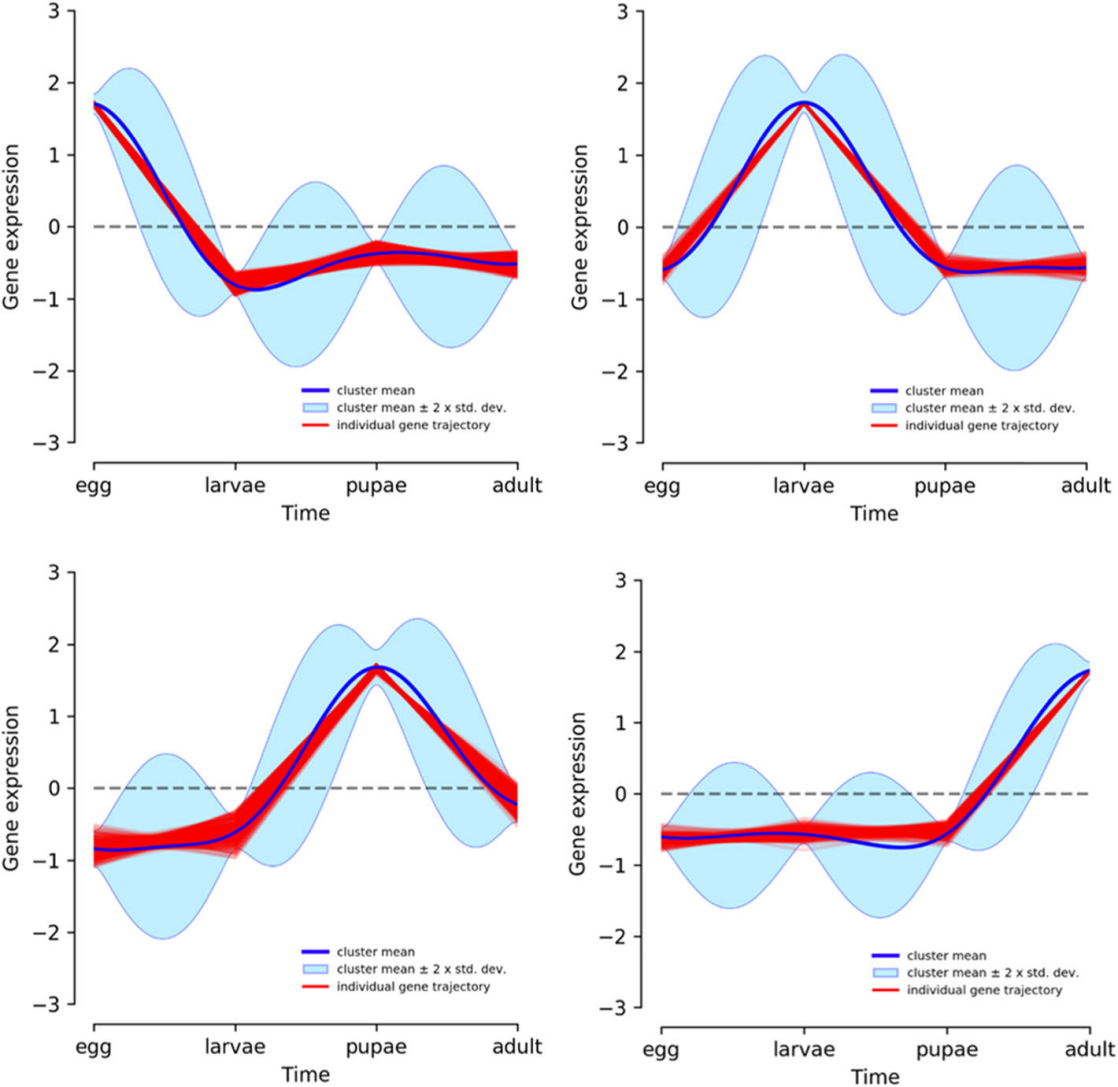
Drichlet process Gaussian process (DPGP) [92] modeling and clustering of gene expression. Gene expression (transcripts per million) was calculated for each of the 4 metamorphotic stages; egg, larvae, pupae, and adult using RSEM [94] and the expression matrix used to determine genes that only peak at the corresponding stage

The olive fruit fly is a holometabolous insect. Egg development lasts 66–70 h in *B. oleae* but this is linearly dependent on temperature [89, 90]. The eggs included in the current study were pooled over 24 h. At this stage the embryos have undergone the maternal-to-zygotic transition, completed blastoderm formation and are just about starting gastrulation [89]. The larva is a specialized feeding stage and the most destructive stage to the olives. The larvae accumulate mass over 10–14 days and undergo successive rounds of molting where the old cuticle is shed and a new one built in response to a hormone [91] called ecdysone [92]. The 28 larvae used in the current study were pooled over the 3 instar stages although 20 were from Instar 1. The 8–12 days immobile pupa stage is a molecularly controlled and highly dynamic stage where the larval tissues are broken down by apoptosis [93] and adult tissues such as wings emerge. The genes specific to the individual stages in the olive fruit fly have not been elucidated. Here, we picked the top 1100 most variable genes across the four metamorphic stages and performed a principal component analysis (PCA, Fig. 6). The first principal component (PC1, accounting for 38% of the total variation), contrasted the adult stage from the egg, larva, and pupa stages. PC2 (accounting for 33% of the total variation), separated the pupae from the egg and larvae. The first principal component thus captures the huge transcriptional differences between the adult and the other developmental stages. The egg and larvae were co-separated perhaps due to most of our larvae coming from L1 stage. To determine the genes that account for these differences we plotted the ‘circle of correlations’ which suggested highly correlated and exclusive sets of genes expressed at the different developmental stages (black dots in Fig. 6). Indeed, hierarchical clustering showed clear clusters of genes that were only highly expressed at specific stages (Supplementary Figure S16). Temporal gene expression has been suggested to follow a Gaussian distribution [95]. In order to identify the developmental stage-specific genes, we clustered all expressed genes using Dirichlet process Gaussian process (DPGP) [96] which jointly models data clusters with a Dirichlet process and temporal dependencies with Gaussian processes. From this, we identified 7086 genes whose expression peaks at different stages, suggesting specific roles for these genes during defined developmental periods (Fig. 7, see Supplementary Table S13 for the genes and corresponding *D. melanogaster* BLAST hits).

Enrichment analysis was performed on the genes in each category using gProfiler [97] and ranked by the adjusted *p*-value. The most enriched GO terms in the egg (Supplementary Figure S17A) were metabolic, biosynthesis, and developmental processes. The most enriched GO terms in the larvae (Supplementary Figure S17B) were chitin metabolic processes, cuticle development, body morphogenesis. For the pupae stage, the most enriched processes were development and morphogenesis related; e.g. multicellular organism development, tube development, imaginal disc morphogenesis, eye development (Supplementary Figure S17 C). We provide a list of the enriched processes in each stage in Supplementary Table S14.

## Discussion

We have assembled the whole genome of *Bactrocera oleae* using both short-read (paired-end and mate-pair approaches), long-read, and linked-reads technologies which encompass all the currently available next generation sequencing technologies. The linked-reads approach generated the most contiguous assembly with a scaffold N50 of 2.16 Mb. This was then chosen as a backbone for scaffolding and gap-closing, which more than doubled the genome contiguity; N50 increased to 4.69 Mb. The final assembly (GenBank accession GCA_001188975.4) is one of the most highly contiguous Tephritidae assembly in the NCBI catalogue (see Supplementary Figure S18 for some comparisons). We were able to achieve this because the laboratory strain of the olive fruit fly used for genome sequencing has low heterozygosity due to (i) low level of natural polymorphism, (ii) significant bottleneck during colonization, and (iii) long period of laboratory rearing (over 45 years) without any admixture. This significantly reduces the ambiguities during the assembly process thus increasing contiguity. Further, we were able to extract high molecular weight DNA that was used to prepare long-read and linked-read sequencing libraries. Due to technological advancements, the library preparation and sequencing costs of short- and long-read technologies is converging at USD 20–40 per Gb [98]. Linked-reads library preparation adds another USD ~ 475 per sample. Considering large genome sequencing efforts like i5k, we find that de novo genome assembly using the linked-reads approach followed by scaffolding and gap-closing using long-reads provides high quality assemblies. For small organisms, like flies, that would be difficult to yield micrograms of high-quality DNA from a single organism in order to reduce heterozygosity and thus increase contiguity and accuracy during genome assembly the linked-reads approach is highly suited. The Hi-C library preparation approach [99] has been shown to yield chromosomal length assemblies, but the method relies on the existence of suitable high-quality tissue and a contiguous assembly as input (ideally with an N50 of ~ 1 Mb as for example in the case of applying the Dovetail™ Hi-C and HiRise™ methodology [100]). The Hi-C method and its variant, the ‘Chicago’ method [101], which only requires high molecular weight DNA, can be used whenever possible to increase contiguity [100, 102].

The assembly presented here will enormously boost the understanding of the olive fruit fly’s biology and genome evolution and shed light to its particularities. For example, as a strictly monophagous insect, olive fruit fly larvae restrict their diet to the olive sap, adapting their physiology to a single nutritional resource and relying on intestinal symbionts to supply essential dietary components that are not supplied by the olive fruit [103, 104]. Being consummate specialists, olive fruit fly larvae may restrict and at the same time specialize their defenses to the plant host, the olive fruit. Such adaptations inevitably should be reflected in its genome and these can now be investigated (for review on insect genome adaptation to host plants see [105, 106]).

Dipteran flies typically have 6 diploid chromosomes with an XY heterogametic system [107]. Although the X chromosome largely retains the organization of its autosome ancestor, the Y chromosome undergoes massive gene decay and general degeneration accompanied by accumulation of repetitive sequences [108]. The highly repetitive nature of the Y chromosome makes it the most challenging to assemble in genome sequencing efforts. The Y chromosome is not devoid of genes. Indeed, in *D. melanogaster*, the Y chromosome contains at least 12 genes [35]. However, some of these genes contain megabase size introns and repeats making them difficult to sequence which then necessitates the use of long-read approaches to assemble them [109, 110]. All *D. melanogaster* Y genes have male-specific functions yet only 3 seem to be conserved while 7 were acquired less than 63 Myr ago showing the high rate of gene gain in flies [35]. These factors demonstrate the need to assemble the highly dynamic but critical Y chromosome and identify its genes. Methods of Y chromosome identification include PCR amplification of Y-linked markers as was done for *Anopheles gambiae* [111], bacterial artificial chromosome cloning followed by mapping and sequencing as was done for humans and primates [112] or BLAST search of unmapped scaffolds in assembled genomes as was done for *D. melanogaster* [113]. Newer technologies include BioNano maps [114], Hi-C [115] and fluorescence in situ hybridization (FISH) of markers. Another method uses flow cytometry sorting to enrich for the Y chromosome followed by short and long read sequencing and RNA-seq [116]. All these methods have challenges in cost or applicability. Here, we adopted the chromosome quotient method that has been used to successfully identify Y-chromosome sequence in *Anopheles stephensi* and *A. gambiae* [49]. We used our extensive dataset including female WGS, male WGS, and our contiguous male genome assembly to apply the Chromosome Quotient method and identify putative Y and X chromosome scaffolds. Indeed, we assembled 3.9 Mb of *B. oleae* Y chromosome and 6 Mb of X chromosome. We used sequences from these putative Y chromosome scaffolds to design PCR primers and thus experimentally confirmed the amplification of male-specific fragments. The sequence of these scaffolds amounts to 1.7 Mb and provides a valuable resource for Y chromosome gene identification. Previously, 700 kb of Y chromosome was identified for *Bactrocera tyroni* using genotype-by-sequencing data and whole-genome resequencing [117]. Y chromosomes, however, remain difficult to assemble. The *D. melanogaster* Y chromosome is estimated at 40 Mb but only 4.2 Mb is assembled into contigs/scaffolds (Flybase release r6.28), and only recently an improved assembly yielded a total of 14.6 Mb of Y-linked sequence [118]. Importantly, the *B. oleae* Y chromosome is home to the male sex determining factor that is responsible for initiating the sexual determination molecular cascade in Tephritidae and had remained elusive thus far. The male sex determining factor, *MoY* (maleness on the Y) has recently been discovered in *Ceratitis capitata* and *Bactrocera oleae* [38], and indeed, the gene is well captured in our PCR confirmed Y chromosome assembly (scaffold LGAM02015747 in GCA_001188975.4 assembly). Work is underway to identify other genes on *B. oleae* Y chromosome which will provide critical information to our understanding of Tephritidae Y chromosome evolution and factors contributing to the male phenotype.

## Methods

### Breeding of the insects

The olive fruit fly, *Bactrocera oleae,* ‘Demokritos’ strain, that is considered in this study was originally sourced from the Nuclear Research Centre in Athens, Greece where it has been maintained for over 45 years. We have maintained this strain in our laboratory for over 15 years with no wild flies added since then, hence the strain has maintained a genetic uniformity. Olive flies were reared in appropriate holding cages at 25 ± 1 °C, 60 ± 10% relative humidity and 14 L: 10D cycles according to the conditions described in [119]. *B. oleae* individuals were immediately sexed upon their emergence and separated until DNA extraction.

### Genomic DNA preparation

High molecular weight (HMW) *B. oleae* genomic DNA was extracted separately from virgin male and female adult flies following the ‘nuclei DNA extraction’ procedure described in Zhang et al. [120]. Generally, whole body insects were frozen in liquid nitrogen and ground in a mortar with pestle into fine powder. The DNA was extracted with phenol/chloroform, precipitated with ethanol and resuspended in TE buffer (10 mM Tris-HCl, 1 mM EDTA, pH 8).

### Library preparation

To generate the paired-end libraries, Illumina TruSeq DNA library preparation kit was followed together with genomic DNA extracted from both male and female flies. The DNA was sequenced using Illumina HiSeq sequencers. The mate-pair libraries were prepared following the Nextera mate-pair library preparation kit from Illumina according to manufacturer instructions. The target sizes for the libraries were: 3 kb, 5 kb, and 10 kb. Pacific Biosciences Inc. (PacBio, California, USA) DNA libraries were prepared following the 20 kb Template Preparation protocol and SMRTbell Template Prep Kit 1.0 using 7.5 μg of DNA and then sequenced on the RSII sequencer. Oxford Nanopore genomic library preparation protocols SQK-MAP 006, SQK-NSK007, and SQK-LSK108 were followed using 5 μg of HMW DNA. DNA was sequenced on the MinION. To generate the linked-reads libraries, HMW DNA was sent to 10x Genomics (CA, USA) where the library preparation was performed. Final library size was sequenced on one lane of an Illumina HiSeq XTen sequencer as 150 bp paired-end generating a total of 414 million reads or 125 Gb. The mate-pair, PacBio, ONT, and linked-reads libraries were prepared using genomic DNA extracted from male insects only.

### RNA extraction and sequencing

A total of 12 RNA samples were collected from different developmental stages and tissues (Supplementary Table S9). The sample sources included adult flies, embryos (eggs), larvae, pupae as well separate tissues including heads, legs, ovipositor, testes, sex organs, and thorax. Total RNA was extracted using the Trizol method and quality-checked as previously described [28]. The TruSeq stranded library preparation protocol (Illumina) was followed by 100 bp paired-end sequencing with Hiseq 2000/2500 sequencers (Illumina).

### Genome size and heterozygosity estimation

The frequency of k-mers of length = 23 bases was calculated using BBMAP [57] followed by genome size estimation using GenomeScope [58].

### Genome assembly

The initial assembly was generated using both male and female paired-end reads. The reads were combined and assembled together using a short-read assembler Ray [41] which was run with a range of kmer values (see Supplementary Table S1). This short-read assembly was then scaffolded with three mate-pair libraries (see Supplementary Table S1) using SSPACE (version 3.0) [42]. As a final step, PacBio reads were used to fill the sequence gaps left behind by the scaffolding process. This assembly was filtered for scaffolds with more than 10X of average Illumina coverage and a minimum length of 500 bp and submitted to NCBI (GenBank assembly accession: GCA_001188975.2). To generate an ONT based assembly, ONT sequence reads were used for de novo genome assembly of the olive fly using Canu [121]. This assembly was named ONT-only. To assemble a hybrid long-read assembly, reads generated from ONT sequencing for the olive fly and those generated from PacBio sequencing were combined and used to generate a separate hybrid assembly (named ONT-PacBio) using Canu (version 1.5).

### Optimization and de novo genome assembly using linked-reads

The Supernova assembler was used to develop the de novo linked-reads assembly. Several rounds of optimization were performed by changing the number of partitions and coverage required to give the most contiguous assembly (as measured by the assembly NG50, assuming a genome size of 320 Mb [23]). We finally selected the genome assembly with 74X coverage and 540,000 barcodes per partition. The resulting genome was analyzed using Quast [43] using default parameters. This genome was named 10x-only.

### Assembly polishing with Pilon

The 387 million Illumina paired-end sequencing reads (yielding ~100X coverage of the olive fly genome) derived from the 10x Genomics experiment was used to correct all assemblies. Reads were aligned to the genomes using BWA-MEM and resulting alignment files processed using Pilon [122]. The polishing was performed in two rounds to derive the polished assemblies. Pilon was run with default parameters. Due to inherent errors in long-read derived genome assemblies, the uncorrected and error-corrected versions of these assemblies were aligned to the assembly derived from 10x Genomics data, using MUMmer [123] (version 3.23) to determine alignment identity. The parameters used were “-l 100 -c 500 -maxmatch”. The ‘.delta’ output was analyzed with dnadiff (part of MUMmer software) to determine the average alignment identity.

### Identification of symbiont derived sequences

In the first approach, we mapped raw reads (SRX5578411 and SRX5557611) and the male genome assembly (GCA_001188975.4) of the olive fruit fly to reference genomes of Wolbachia, Spiroplasma, and Cardinium using MIRA v4.0 and bowtie2. For the Wolbachia mapping exercise we used complete and draft genomes that were publicly available (4688 contigs in total) as reference sequences. For the Spiroplasma mapping we used the following complete genomes: (a) *Spiroplasma chrysopicola* DF-1, (b) *Spiroplasma syrphidicola* EA-1, complete genome, (c) *Spiroplasma taiwanense* CT-1, complete genome, (d) *Spiroplasma diminutum* CUAS-1. For Cardinium, the Cardinium endosymbiont cEper1 of *Encarsia pergandiella* was used as a reference genome. In the second approach, we downloaded 235,684 complete and draft genomes that have been deposited to NCBI (June 2019). These sequences were used as a custom BLAST database in order to identify bacterial sequences that have been filtered into the assembly of the *B. oleae* genome. Blast results were visualized using BLASTGrabber v.2.

### Benchmarking universal single-copy Orthologs (BUSCOs) analysis

Assembly completeness (genome or transcriptome) was assessed by querying the presence of orthologous sets of evolutionarily conserved genes termed Benchmarking universal single-copy orthologs (BUSCOs) [56] from 4 different phylogenetic lineages; Eukaryota, Arthropoda, Insecta, and Diptera. First, datasets for the 4 different lineages were downloaded from busco.ezlab.org. The assemblies were then successively queried for the presence of each lineage specific BUSCO using the BUSCO software (version 2.0.1).

### Y and X chromosome identification

In order to find putative X or Y chromosome scaffolds we used the Chromosome Quotient method [49] which calculates the median ratio of female to male coverage for each scaffold. The resulting quotient values will cluster around zero, one or two for Y, autosome or X scaffolds respectively. Before aligning the reads, repeats are masked from the assembly using RepeatMasker. We aligned 40x coverage of male and female reads to a hard-masked version of the assembly and for each set, we calculated the depth at each base for all scaffolds. We further filtered out positions with less than 10x of male coverage to ensure a minimum of evidence from male DNA.

### Validation of Y-chromosome specific scaffolds

Putative Y-derived scaffolds were validated through standard PCR and real-time quantitative PCR (RT-qPCR). Specifically, DNA was extracted from three pools of virgin male and female insects each one containing 10 insects. Eighty-five pairs of primers were designed using the Primer3 [124]. PCR reaction was carried out in a final volume of 20 μl, using 1.5 mM MgCl_2_, 1x PCR reaction buffer, 1 Unit Taq DNA polymerase (Bioline, London, UK), 0.35 pmol of each forward and reverse primers and 0.8 mM dNTPs. The amplification conditions were as follows: 94 °C 4 min; 94 °C 30 s, 55 °C 30 s, 72 °C 2 min for 30 cycles; 72 °C 5 min. PCR products were identified by 1–1,5% agarose gel electrophoresis. RT-qPCR was carried out in a final volume of 15 μl, using 1 μl from a 1:10 dilution of the cDNA template, 2X SYBR Select Master Mix (Applied Biosystem) and 300 nM of each primer. The amplification conditions were: polymerase activation at 50 °C for 2 min, DNA denaturation step at 95 °C for 4 min, followed by 50 cycles of denaturation at 95 °C for 10 s, annealing/extension and plate-read at 55 °C for 20 s and finally, a step of melting curve analysis at a gradual increase of temperature over the range 55 °C to 95 °C. The reactions were carried out on a Bio-Rad Real-time thermal cycler CFX96 (Bio-Rad, Hercules, CA, USA) and data were analyzed using the CFX Manager™ software. All PCR reactions were performed in triplicate (i.e., three technical replicates).

### Cloning of probe sequences for in situ hybridization

Specific primers were designed using Primer3 to amplify segments of scaffolds for which there was no previous mapping information available. The probe amplification was carried out in a 20 μl PCR reaction volume using 1.5 mM MgCl_2_, 1X PCR reaction buffer, 1 unit Taq DNA polymerase (Bioline, London, UK), 0.35 pmol of each forward and reverse primers and 0.8 mM dNTPs. The amplification conditions were as follows: 94 °C 4 min; 94 °C 30 s, Tan* °C 30 s, 72 °C extension time for 30 cycles; 72 °C 5 min. The PCR products after electrophoresis were gel purified by the Wizard® SV Gel and PCR Clean-Up System (Promega, Madison, WI, USA) following the manufacturer’s instructions, ligated into TA cloning vector pTZ57R/T (Thermo Scientific InsTAclone PCR Cloning Kit) and finally used to transform electrocompetent *E. coli* DH5α cells according to standard procedures. The recombinant plasmid DNA was finally isolated with the use of the Promega Wizard Plus Minipreps DNA Purification System according to the supplier’s instructions.

### Chromosome preparations and in-situ hybridization

Polytene chromosome spread preparations were obtained from the salivary glands of third instar larvae and young pupae (1–2 days old) [125]. The random priming method was used to generate the digoxigenated dUTP (Dig-11dUTP) labelled probes. Hybridization was performed at 62 °C and signal detection was performed using the DIG DNA Labeling and Detection kit (ROCHE Diagnostics, Mannheim, Germany) according to Drosopoulou et al. [125]. Two to three preparations were hybridized with each probe, and at least ten well spread nuclei per preparation were analyzed. The pretreatment of chromosome preparations, hybridization, detection and image analysis are described in detail in [125, 126]. The hybridization sites were identified according to the available polytene chromosome maps [52, 54].

### Transposable element (TE) identification

We used the PiRATE [67] pipeline for TE identification. Starting with the assembled genome we used the “similarity-based” tools (RepeatMasker [127]; TE-HMMER), “Structural-based” tools (MITE Hunter [128], HelSearch [129], LTR Harvest [130], SINE-Finder [131], MGEScan-LTR [132]), and “Repeatitiveness-based” tools (TEdenovo [69], RepeatScout [133]). TEs overlapping by 100% of a larger element were removed using CD-HIT-est [134] and the remaining TE classified using PASTEC [69]. Following TE library generation, the sequences were BLAST’ed. against the *B. oleae* proteome and best hits with > 50% alignment identity, > 100 nucleotide alignment and Evalue > 0.001 were removed from the TE library. Finally, the library was used to annotate the genome using TEannot [70].

### De novo transcriptome assembly

We used a pipeline developed following the protocol described in Haas et al. [135] and mostly based on the Trinity assembly software suite [77]. Normalization was performed in order to reduce memory requirement and decrease assembly runtime by reducing the number of reads, using the Trinity normalization utility [77] inspired by the Diginorm algorithm [136]. Haas et al. [135] showed that normalization results in full-length reconstruction to an extent approaching that based on the entire read set. In addition, each assembly contig and component were analyzed using the Trinotate annotation pipeline. We also performed Trinity genome-guided transcriptome assembly.

### Genome feature and functional annotation

Feature annotation to generate the official *B. oleae* gene model set (OGS) was completed using the JAMg annotation pipeline [78] as previously applied [44]. Briefly, the pipeline involved repeat masking using RepeatModeler (v1.0.8), RepeatScout (v1.0.5), and RepeatMasker (v4–0-6), and the generation of transcriptome database for model training using Augustus. A separate prediction was run using GeneMark-ES (4.38). As EvidenceModeler removes the UTR and alternative transcripts predicted from Augustus, we used PASA to update these models and create the final JAMg OGS. Functional annotation of *B. oleae* gene models predicted by the JAMg annotation pipeline was performed using Blast2GO [80] included in OmicsBox version 1.1.78. Each protein (or the longest protein for multi-isoform genes) was Blast-searched again the Swiss-Prot database (Evalue, 1e-4) with output format 15 selected. XML Blastp results and sequences were imported into Blast2GO [80] and used to retrieve domain and motif signatures via Interproscan [81] analysis followed by identification of gene ontology (GO) terms via mapping and assignment of GO terms to sequences through functional annotation.

### Phylogenetic classification

We used Prot-SpaM [83] to infer pairwise distances of 19 species using complete proteomes. A phylogenetic tree was generated using *Neighbour-Joining* algorithm [84] implemented in T-REX [85] and viewed using iTOL [86].

### Identification of orthologous proteins

Orthologs among *D. melanogaster, M. domestica, C. capitata, Z. cucurbitae, B. dorsalis* and *B. oleae* were identified using OrthoFinder [87]. Supplementary Table S12 contains all orthogroups and the proteins from each species that belong to respective orthogroups.

### Principle component analysis and hierarchical clustering

Gene expression (transcripts per million, TPM) was calculated for each of the 4 metamorphotic stages; egg, larvae, pupae, adult using RSEM [94] and used to calculate gene z-score on the log transformed TPM. Principle component analysis on the 1100 topmost variable genes among the stages was performed by the “prcomp” function then plotted by the “biplot” function both of R statistical software.

### Temporal clustering of developmental stage-specific genes

The expression matrix (transcripts per million, TPM) filtered for genes that were not expressed at any of the stages was used as input to Dirichlet process Gaussian process (DPGP) [96] to cluster genes with similar expression profiles. Clusters of Genes in clusters that peak at either of the 4 metamorphotic stages; egg, larvae, pupae, adult were combined and used in gene ontology enrichment analysis using gProfiler [97].

## Supplementary information

**Additional file 1.** Supplementary materials.

**Additional file 2: Table S1.** Sequencing libraries used, sequence results, and kmer optimization for the GCA_001188975.2 assembly. **Table S2.** Comparison of assembly quality of the 3 main assemblies. **Table S3.** Assembly statistics for 9 different assemblies generated. **Table S4.** Comparison of assembly quality of the 3 Y chromosome assemblies. **Table S5.** Sequences of the primers used for the validation of Y-chromosome specific scaffolds. **Table S6.** Mapping positions and primers used to generate 9 new *B. oleae* DNA markers in this study. **Table S7.** Scaffold/contig localization on *B. oleae* chromosomes. **Table S8.** Distribution of the *B. oleae* Transposable elements in the genome assembly. **Table S9.** Samples and tissues used for the transcriptome sequencing and assembly. **Table S10.** Summary of the Trinity transcriptome generated from sequencing all the tissues in Supplementary Table S7. **Table S11.** Assessment of the completeness of the Trinity de novo transcriptome assembly. **Table S12.** Orthologous genes among 6 closely related insects. **Table S13.** Genes that peak at different metamorphic stages. **Table S14.** Gene ontology enrichment analysis for genes that peak at different metamorphic stages. **Table S15.** Datasets submitted to NCBI and their corresponding accession numbers and description. **Table S16.** Sources of proteomes and genomes used in Supplementary figures.

**Additional file 3: Figure S1.** Schematic of the method used to generate the main assembly reported. **Figure S2.** Genome size and heterozygosity estimation. **Figure S3.** Contig length at different Nx values for assemblies in Supplementary Table S3. **Figure S4.** Contiguity plot generated using Quast. **Figure S5.** Contig length at different Nx values for assemblies in Supplementary Table S4. **Figure S6.** Plot showing Y chromosome scaffolds/contigs identified in 3 different assemblies (Supplementary Table S4). **Figure S7.** Total length of scaffolds that were localized to each polytene chromosome and XY chromosomes. **Figure S8.** Alignment rates of RNA-seq reads from 12 different *Bactrocera oleae* datasets (see Supplementary Table S9). **Figure S9.** Complete Basic Universal Single Copy Orthologs (BUSCOs) identified in genome assemblies (Supplementary Table S3). **Figure S10.** Schematic of the PiRATE pipeline. **Figure S11.** Histogram of transcripts read lengths. **Figure S12.** Percentage of Arthropoda Basic Universal Single Copy Orthologs (BUSCOs) captured in 19 arthropod transcriptomes. **Figure S13.** Number of JAMg predicted *B. oleae* genes located on the scaffolds assigned to polytene element. **Figure S14.** Gene ontology (GO) classification of *B. oleae* JAMg predicted proteins. **Figure S15.** Detailed orthogroup distribution. **Figure S16.** Hierarchical clustering of 1100 most variable genes among the 4 metamorphotic stages. **Figure S17.** Most significantly enriched gene ontology (GO) terms among genes that only peak during development. **Figure S18.** Contig length at different Nx values for assemblies of selected insects.

## Data Availability

The Genome sequence has been submitted to NCBI with GenBank accession number GCA_001188975.4. All raw reads and RNA-seq data have been submitted to SRA using the study number PRJNA288990. See Supplementary Table S15 for SRA accession numbers for each dataset.

## Abbreviations

SIT: Sterile Insect Technique
ONT: Oxford Nanopore Technologies
BUSCO: Basic Universal Single Copy Orthologs
TGS: Third generation Sequencing
PacBio: Pacific Bioscience
ISPRA: Italian National Institute for Environmental Protection and Research
kb: kilobase(s)
Mb: Megabase(s)
Gb: Gigabases
ng: nanogram(s)
NCBI: National Center of Biotechnology Information
Myr: Million years
PCR: Polymerase chain reaction
vs: versus
N50: Scaffold/contig length at which 50% of the total genome length is contained in scaffolds/contigs of that size or longer when all scaffolds/contigs are ordered from longest to shortest
NG50: N50 except the genome size is fixed and not dependent on sum of scaffolds/contigs
L50: Total number of scaffolds/contigs needed to reach N50
LG50: Total number of scaffolds/contigs needed to reach NG50
rpm: rounds per million
ml: milliliter
μg: microgram
mM: millimolar
EDTA: Ethylene di-amine tetra-acetic acid
nM: nanomolar
pM: picomolar
μl: microlitre
HMW: High molecular weight
OGS: Official gene set

## Footnotes

1 George Orwell, Animal Farm
