# Supplementary material for: De novo assembly of the olive fruit fly (Bactrocera oleae) genome with linked-reads and long-read technologies minimizes gaps and provides exceptional Y chromosome assembly
Source: BMC Genomics. 2020 Mar 30;21:259. doi: 10.1186/s12864-020-6672-3 (PMC7106766; doi:10.1186/s12864-020-6672-3)
Supplement: Supplementary file 1 — Additional file 1. Supplementary materials. [file 12864_2020_6672_MOESM1_ESM.docx]

**Materials and methods**

**Breeding of the insects**

The olive fruit fly, *Bactrocera oleae,* ‘Demokritos’ strain, that is considered in this study was originally sourced from the Nuclear Research Centre in Athens, Greece where it has been maintained as a lab strain for over 45 years. This strain has been maintained in our lab (Laboratory of Molecular Biology and Genomics at the Department of Biochemistry and Biotechnology, University of Thessaly) for over 15 years with no wild flies added. Hence the strain has maintained a genetic uniformity. Olive flies were reared in appropriate holding cages at 25 ± 1˚ C, 60 ± 10% relative humidity and 14 L: 10D cycles according to the conditions described in [1]. *B. oleae* individuals were immediately sexed upon their emergence and separated until DNA extraction.

**Genomic DNA preparation**

High molecular weight (HMW) *B. oleae* genomic DNA was extracted separately from virgin male and female adult flies following the ‘nuclei DNA extraction’ procedure described in Zhang et al. [2]. Generally, whole body insects were frozen in liquid nitrogen and ground in a mortar with pestle into fine powder. Homogenization buffer (HB, 1.5 ml) was added to resuspend the powder and the homogenate was centrifuged at 1,400 rpm for 8 minutes. Another 1.5 ml 1X HB containing proteinase K (to a final concentration of 200 μg/ml) was added to the recovered supernatant and the homogenate was further incubated at 45˚C for 1 hour. The DNA was extracted with phenol/chloroform, precipitated with ethanol and resuspended in TE buffer (10 mM Tris-HCl, 1 mM EDTA, pH 8).

**DNA quality and quantity assessment**

DNA quality was assessed using a NanoDrop ND-2000 (Thermo Fischer Scientific, USA) and genomic ScreenTape on the TapeStation 2200 (Agilent, USA) to determine the profile. DNA profiles were also determined by running samples on Pulse Field Gel Electrophoresis (PFGE) Pippin Pulse system (Sage Sciences, USA). The concentration of DNA was assessed using the dsDNA HS assay on a Qubit fluorometer (Thermo Fischer Scientific, USA).

**Illumina Mate-pair library preparation**

The ‘Nextera mate-pair library preparation kit’ from Illumina was used following manufacturer instructions. The target sizes for the libraries were: 3 kb, 5 kb, and 10 kb. Briefly, HMW genomic DNA was tagmented with a mate-pair transposome and then circularized and sheared followed by bead-purification. The purified molecules (representing the mate-pairs) were treated with TruSeq end repair, dA-tailing, and adapter ligation reagents followed by PCR amplification and sequencing. Only male DNA was used for mate-pair library preparation.

**Illumina paired-end library preparation**

Paired-end libraries were generated using Illumina TruSeq DNA library preparation kit following manufacturer instructions. Briefly, 1 µg of genomic DNA was fragmented to 300 – 400 bp using Covaris (Covaris, USA). The fragmented DNA was end-repaired, dA-tailed, adapter ligated, followed by PCR amplification. The PCR amplified DNA was normalized and sequenced using Illumina HiSeq sequencers.

**PacBio DNA library preparation and sequencing**

The DNA libraries were prepared following the Pacific Biosciences Inc. (PacBio, California, USA) 20 kb Template Preparation Genomic protocol and SMRTbell Template Prep Kit 1.0. Briefly, 7.5 µg of HMW DNA was fragmented using Covaris g-TUBE (Covaris, USA) following manufacturer’s instructions for a target size of 20 kb. The sheared DNA was then size selected on a BluePippin system (Sage Science Inc., USA) using a cutoff range of 7 kb or 15 kb to 50 kb. DNA damage repair, End Repair, and SMRT bell adapter ligation were performed on the size-selected DNA following protocol. The sequencing primer was annealed at a final concentration of 0.8333 nM and the polymerase was bound at 0.500 nM. The libraries were sequenced on a PacBio RSII instrument at a loading concentration (on-plate) of 60 pM to 100 pM using the MagBead loading protocol, DNA sequencing kit 2.0 and 4.0, SMRT cells v3 with 3- or 4-hours movies. A total of 21 SMRT cells were used for sequencing.

**10x Genomics library preparation**

The current configuration of the 10x Chromium system (DNA input amount, number of partitions, and sequencing depth) are optimized for the human genome and need to be modified for smaller genomes [3]. The key features of our sample preparation were the extraction of high molecular weight DNA and size selection for DNA molecules > 40 kb reads (Blue Pippin, SAGE Biosciences). The profile of HMW DNA (10 µg) from male flies was assessed by pulse field gel electrophoresis (PFGE) on a Pippin Pulse (SAGE Biosciences) 0.75% agarose gel. DNA was also quantified by running on an Agilent Tapestation Instrument whereby, it was estimated that approximately 40 % of the DNA was > 48.5 kb and at 46 ng/µl. HMW DNA (> 30 kb) was purified using a Pippin HT instrument (SAGE Biosciences) which yielded a concentration of 3.46 ng/µl and a total yield of 86.5 ng. This DNA was sent to 10x Genomics (USA) where the library preparation was performed. Final library size was at 696 bp and was sequenced in one lane of an Illumina HiSeq XTen sequencer as 150 bp paired-end generating a total of 414 million reads or 125 Gb.

**Oxford Nanopore Technologies (ONT) DNA library preparation and sequencing**

**Library preparation**

Oxford Nanopore genomic library preparation protocols SQK-MAP006, SQK-NSK007, and SQK-LSK108 were followed. Briefly, 5 μg of HMW DNA was treated with NEBNext FFPE DNA repair module (New England BioLabs, UK) to perform DNA repair. DNA was purified using Agencourt AMPure XP beads (Beckman coulter, USA). Purified DNA was treated with NEBNext Ultra II end repair/dA-Tailing module (New England BioLabs, UK), to perform end-repair and dA tailing, followed by DNA purification. Finally, Oxford Nanopore adapters, and tether were ligated to end repaired DNA using Blunt/TA ligase Master Mix (New England Biolabs). All steps were performed using DNA Lobind 1.5 ml tubes to minimize DNA loss. The resulting pre-sequencing mix (PSM) was further quality-checked using TapeStation and quantified using Qubit fluorometer.

**MinION sequencing**

Three flowcell chemistries were used; R7.3 (used with SQK-MAP006 kit), R9 (used with SQK-NSK007 kit), and R9.4 (used with SQK-LSK108 kit). Sequencing was performed using the MinION following ONT sequencing protocol. Briefly, flowcells were warmed to room temperature for at least 30 minutes. Platform quality control was performed by running the CTC_platform_QC.py script. A minimum of 350 active pores (out of 512) in group 1 was considered good enough. Library was loaded, and the 48-hour sequencing script run.

**Oxford Nanopore signal-basecalling**

Base-calling was performed after the sequencing run. Signal files generated with R7.3 chemistry were basecalled using Metrichor while those generated with R9 and R9.4 chemistry were basecalled using Albacore (Oxford Nanopore).

**RNA extraction and sequencing**

A total of 15 RNA samples were collected from different developmental stages and tissues (Supplementary Table S7). The sample sources included adult flies, embryos (eggs), larvae, pupae as well separate tissues including heads, legs, ovipositor, testes, sex organs and thorax. Total RNA was extracted using the Trizol method and quality-checked as previously described [4]. The TruSeq stranded library preparation protocol was followed (Illumina) followed by 100 bp paired-end sequencing with Hiseq 2000/2500 sequencers (Illumina). Base quality is encoded in phred 33. Read trimming and clipping were performed using the Trimmomatic software [5].

**Genome size and heterozygosity**

The frequence of k-mers of length=23 bases was calculated using BBMAP followed by genome size estimation using GenomeScope [6]

**Illumina Data analysis**

***De novo* genome assembly from Illumina**

Using Trimmomatic [5], reads were first trimmed from the 3' end to have a phred score of at least 30. Illumina sequencing adapters were then removed from the reads, and all reads were required to have a length of at least 65 bases. The male and female reads were combined and assembled together using a short-read assembler Ray [7] which was run with a range of kmer values (see Supplementary Table S1). According to kmergenie [8] the best kmer for our mixed dataset was k51. However, slightly smaller kmers produced similar assemblies. In the end k41 was chosen for having produced the largest contig. Both male and female reads were also assembled independently but produced fewer good assemblies than mixed-reads assembly (results not shown).

The mate-pair reads were first trimmed using NextClip [9] and placed in the right orientation with the second read reverse complemented. The effective library size was then estimated from the data by aligning the mate-pairs to the male assembly with BWA-MEM [10] and calculating the distance between the reads from alignments to the same contig using sam_bam2tab.pl tool of SSPACE. The final short read assembly was then scaffolded using the three mate-pair libraries (see Supplementary Table S1). Scaffolding was performed using SSPACE (version 3.0) [11] using the following parameters;

perl ~/SSPACE_Standard_v3.0.pl -l lib.txt -s assembly.fastq -k 5 -a 0.7 -x 0 -p 1 -b outputname -g 1 -T 12

As a final step, PacBio reads were used to fill the sequence gaps left behind by the scaffolding process resulting in a decrease in gap bases from 44,015,646 bases to 27,804,509. This assembly was filtered for scaffolds with more than 10X of average Illumina coverage and a minimum length of 500 bp and submitted to NCBI (GenBank assembly accession: GCA_001188975.2).

**Optimization and d*e novo* genome assembly using linked-reads**

In the 10x Genomics system, individual DNA molecules are encapsulated in an oil emulsion together with barcoded primers which indicate reads that originated from the same partition. A total of 414,094,826 reads were generated from which 22,793,359 barcodes (partitions) were identified. However, 13,528,431 barcodes/partitions were rejected as low quality leaving 9,264,928. We then corrected the barcode sequences reducing the number of partitions to 3,700,604 containing 395,344,827 reads. The partitions were further filtered to retain only those that had > 10 reads, leaving 1,443,720 partitions containing 389,830,635 reads. Each read was then assigned to its partition. The Supernova assembler was then used to develop the *de novo* assembly. Several rounds of optimization were performed by changing the number of partitions and coverage required to give the most contiguous assembly (as measured by the assembly NG50). We finally selected the genome assembly with 74X coverage and 540,000 barcodes per partition. The resulting genome was analyzed using Quast [12]. This genome was named 10x-only

**Long-read (ONT and PacBio) *de novo* genome assembly**

FastA reads for sequence files basecalled with Metrichor were extracted using NanoOK [13] while Albacore generated FastA reads by default. Reads’ metadata such as quality of reads and run performance were extracted from read files using custom scripts. To generate an ONT only based assembly, sequence reads from all flow cells were combined and used for *de novo* genome assembly of the olive fly using Canu [14] (version 1.5). Since previous work [15] had estimated the genome size of the olive fly to be 322 Mb, Canu assembly was performed with “genomeSize=320m” parameter as follows;

useGrid=false -p ONT-only -d dir genomeSize=320m -nanopore-raw ont.fasta ovlErrorRate=0.12 overlapper=mhap utgReAlign=true

The resulting assembly was analyzed by both custom scripts and Quast and named ONT-only. The ONT-only assembly had 2,473 contigs with a total size of 384 Mb. The assembly N50 was 258 kb and the assembly L50 was 408. The largest contig was 4.38 Mb, and the assembly was generated without any ‘N’s and thus reflects true contigs and not scaffolds. We did not attempt to make a PacBio only assembly due to low coverage.

To assemble a hybrid long-read assembly, reads generated from ONT sequencing for the olive fly and those generated from PacBio sequencing were combined and used to generate a separate hybrid assembly (named ONT-PacBio) using Canu (version 1.5) with the following parameters;

useGrid=false -p ONT-PacBio -d dir genomeSize=320m ovlErrorRate=0.12 overlapper=mhap utgReAlign=true -pacbio-raw pacbio.fastq -nanopore-raw ont.fasta

The ONT-PacBio assembly yielded 1,562 contigs totaling 421.87 Mb with N50 of 697.7 kb, and L50 of just 165 contigs.

**Scaffolding of the 10x-only assembly**

Long reads produced from ONT sequencing were used to scaffold the 10x Genomics assembly to improve its contiguity. Scaffolding was performed with npScarf [16] (version 1.6-11c) which works by first identifying unique (due to low multiplicity) and repetitive contigs or scaffolds in the draft assembly. Long ONT reads are then used to bridge two unique contigs or scaffolds while repetitive sequences are used to close gaps in the scaffolds. In our analysis, scaffolds in the draft assembly were first sorted using default settings of the script jsa.seq.sort, followed by indexing of the sorted file using BWA-MEM [10]. The sorted sequences were then scaffolded using npScarf with the following parameters:

jsa.np.npscarf_e -all –prefix=new_scaffold –bwaThread=45 –seqFile=draf.assembly.fasta –input=ONT.reads.fasta

**Assembly polishing with Pilon**

The 387 million Illumina paired-end sequencing reads (yielding ~100X coverage of the olive fly genome) derived from the 10x Genomics experiment was used to correct all assemblies. Reads were aligned to the genomes using BWA-MEM, and resulting alignment files processed using Pilon [17]. The polishing was performed in two rounds to derive the polished assemblies. Pilon was run with default parameters.

Due to inherent errors in long-read derived genome assemblies, the uncorrected and error-corrected versions of these assemblies were aligned to the assembly derived from 10x Genomics data, using MUMmer [18] (version 3.23) to determine alignment identity. The parameters used were “-l 100 -c 500 -maxmatch”. The ‘.delta’ output was analyzed with dnadiff (part of MUMmer software) to determine the average alignment identity. Error correction of the assembly using Pilon [43] increased alignment identity from 97.92 % to 99.26 %. Error-correction of the ONT-PacBio assembly dramatically increased the number of Dipteran complete ancestral genes captured from 47.2 % to 97.2 %.

**Assessing assembly completion with Benchmarking Universal Single-Copy Orthologs (BUSCOs)**

Assembly completeness was assessed by querying the presence of orthologous sets of evolutionarily conserved genes termed Benchmarking universal single-copy orthologs (BUSCOs) [19] from 4 different phylogenetic lineages; eukaryotes, arthropods, insecta, and diptera. First, datasets for the 4 different lineages were downloaded from busco.ezlab.org. The assembled genomes were then successively queried for the presence of each lineage specific BUSCO using the following BUSCO software (version 2.0.1) parameters:

BUSCO.py -n 10 -i input.genome.fasta -o output -l lineage.busco_odb9 -m geno

**Screening of *B. oleae* Genome Sequence Data for symbiont derived sequences**

**Approach 1**

The raw data and the male genome assembly (GCA_001188975.4) of the olive fruit fly whole genome sequence project that were publicly available were used (SRX5578411 and SRX5557611). These reads were mapped to reference genomes of Wolbachia, Spiroplasma, and Cardinium using MIRA v4.0 and bowtie2. For the Wolbachia mapping exercise we used complete and draft genomes that were publicly available (4688 contigs in total) as reference sequences. For the *Spiroplasma* mapping we used the following complete genomes: (a) *Spiroplasma chrysopicola* DF-1, (b) *Spiroplasma syrphidicola* EA-1, complete genome, (c) *Spiroplasma taiwanense* CT-1, complete genome, (d) *Spiroplasma diminutum* CUAS-1. For Cardinium, the Cardinium endosymbiont cEper1 of *Encarsia pergandiella* was used as a reference genome.

**Approach 2**

We downloaded 235,684 complete and draft genomes that have been deposited to NCBI (June 2019) and these sequences were used as a custom blast database in order to identify bacterial sequences that have been filtered into the assembly of the *B. oleae* genome. Blast results were visualized using BLASTGrabber v.2.

**Y and X chromosome identification**

In order to find putative X or Y chromosome scaffolds we used the Chromosome Quotient method [20] which calculates the median ratio of female to male coverage for each scaffold. The resulting quotient values will cluster around zero, one or two for Y, autosome or X scaffolds respectively. Before aligning the reads, repeats are masked from the assembly using RepeatMasker. Starting with 40X of male and female coverage we independently mapped both read sets to a hard-masked version of the assembly and for each set, we calculated the depth at each base for all scaffolds. We further filtered out positions with less than 10X of male coverage to ensure a minimum of evidence from male DNA. Then for each scaffold we calculated the CQ value for all positions. For Y chromosome we considered only the scaffolds with a CQ of 0. Similarly, we attempted to capture X chromosome scaffolds using a CQ range of 1.9 to 2.1 and a minimum coverage of 10X from male and female reads. we attempted to validate the Y chromosome scaffolds using an orthogonal method; Y chromosome genome scan (YGS) [55]. YGS was run with default parameters. To identify Y chromosome scaffolds, we chose those scaffolds that had 90 % or more of the scaffold with no coverage of female reads.

**Validation of Y-chromosome specific scaffolds**

Putative Y-derived scaffolds were validated through standard PCR and real-time quantitative PCR (RT-qPCR). Specifically, DNA was extracted from three pools of virgin male and female insects each one containing 10 insects. Eighty-five pairs of primers were designed using the Primer3. PCR reaction was carried out in a final volume of 20 μl, using 1.5 mM MgCl_2_, 1X PCR reaction buffer, 1 Unit Taq DNA polymerase (Bioline, London, UK), 0.35 pmol of each forward and reverse primers and 0.8 mM dNTPs. The amplification conditions were as follows: 94 °C 4 min; 94 °C 30 s, 55 °C 30 s, 72 °C 2 min for 30 cycles; 72 °C 5 min. PCR products were identified by 1-1,5% agarose gel electrophoresis.

RT-qPCR was carried out in a final volume of 15 μl, using 1 μl from a 1:10 dilution of the cDNA template, 2X SYBR Select Master Mix (Applied Biosystem) and 300 nM of each primer. The amplification conditions were: polymerase activation at 50 °C for 2 min, DNA denaturation step at 95 °C for 4 min, followed by 50 cycles of denaturation at 95 °C for 10 s, annealing/ extension and plate read at 55 °C for 20 s and finally, a step of melting curve analysis at a gradual increase of temperature over the range 55 °C to 95 °C. The reactions were carried out on a Bio-Rad Real-time thermal cycler CFX96 (Bio-Rad, Hercules, CA, USA) and data were analyzed using the CFX Manager™ software. All PCR reactions were performed in triplicate (i.e., three technical replicates).

## Cloning of probe sequences for *in situ* hybridization

Specific primers were designed using Primer3 to amplify segments of scaffolds for which there was no previous mapping information available. The probe amplification was carried out in a 20 μl PCR reaction volume using 1.5 mM MgCl_2_, 1X PCR reaction buffer, 1 unit Taq DNA polymerase (Bioline, London, UK), 0.35 pmol of each forward and reverse primers and 0.8 mM dNTPs. The amplification conditions were as follows: 94 °C 4 min; 94 °C 30 s, Tan* °C 30 s, 72 °C extension time for 30 cycles; 72 °C 5 min.

The PCR products after electrophoresis were gel purified by the Wizard1 SV Gel and PCR Clean-Up System (Promega, Madison, WI, USA) following the manufacturer’s instructions, ligated into TA cloning vector pTZ57R/T (Thermo Scientific InsTAclone PCR Cloning Kit) and finally used to transform electrocompetent *E. coli* DH5α cells according to standard procedures (Sambrook et al. 1989). The recombinant plasmid DNA was finally isolated with the use of the Promega Wizard Plus Minipreps DNA Purification System according to the supplier’s instructions.

## Chromosome preparations and *in-situ* hybridization

Polytene chromosomes’ spread preparations were obtained from the salivary glands of third instar larvae and young pupae (1-2 days old) [21]. The random priming method was used to generate the digoxigenated dUTP (Dig-11dUTP) labelled probes. Hybridization was performed at 62 °C and signal detection was performed using the DIG DNA Labeling and Detection kit (ROCHE Diagnostics, Mannheim, Germany) according to Drosopoulou et al [21]. Two to three preparations were hybridized with each probe, and at least ten well spread nuclei per preparation were analyzed. The pretreatment of chromosome preparations, hybridization, detection and image analysis are described in detail in [21, 22]. The hybridization sites were identified according to the available polytene chromosome maps [23, 24].

**Transposable element (TE) identification**

We used the PiRATE [25] pipeline for TE identification. Starting with the assembled genome we used the “similarity-based” tools (RepeatMasker [26]; TE-HMMER), “Structural-based” tools (MITE Hunter [27], HelSearch [28], LTR Harvest [29], SINE-Finder [30], MGEScan-LTR [31]), and “Repeatitiveness-based” tools (TEdenovo [32], RepeatScout [33]). We skipped the tools that utilize raw reads to build repeated elements (dna PipeTE [34], RepArk [35], Repeat Explorer [36]). Overlapping TE were removed using CD-HIT-est [37] and the TE classified using PASTEC [32]. Following TE library generation, the sequences were BLAST’ed against the *B. oleae* proteome and best hits with > 50% alignment identity, >100 nucleotide alignment and evalue > 0.001 were removed from the TE library. Finally, the library was used to annotate the genome using TEannot [38].

***De novo* transcriptome assembly**

We used a pipeline developed following the protocol described in Haas et al. [39] and mostly based on the Trinity assembly software suite [40]. Normalization was performed in order to reduce memory requirement and decrease assembly runtime by reducing the number of reads, using the Trinity normalization utility [40] inspired by the Diginorm algorithm [41]. Haas et al. [39] showed that normalization results in full-length reconstruction to an extent approaching that based on the entire read set. In addition, each assembly contig and component were analyzed using the Trinotate annotation pipeline. We also performed Trinity genome-guided transcriptome assembly.

**Genome feature and functional annotation**

Feature annotation to generate the official *B. oleae* gene model set (OGS) was completed using the JAMg annotation pipeline [42] as previously applied [43]. Briefly, the pipeline involved: repeat masking using RepeatModeler (v1.0.8), RepeatScout (v1.0.5), and RepeatMasker (v4-0-6); the generation of *de novo* and genome-guided transcriptomes using Trinity (v2.8.5); the creation of PASA (v2.0.2) [44] transcriptome database; the generation of a set of transcriptome-based, full-length, putative genes models with high confidence; the alignment of all RNA-seq data (using GSNAP May 2019) and Nanopore cDNA (using minimap2 v2.17-r943-dirty) to the genome to create evidence tracks for mRNA coverage and intron exon splice junctions; poly-A signals as derived from the PASA database. These data were complemented with the official gene set (OGS) of the RefSeq annotation of the previous version of the *B. oleae*. The data where then provided to Augustus to first train the HMM and then – using the high confidence transcriptome-based gene models - derive relative weights for each evidence. A separate prediction was run using GeneMark-ES (4.38). A single gene model track was predicted using EvidenceModeler using the results from Augustus, GeneMark-ES, PASA, protein alignments using BLASTp vs SwissProt, transcript alignments of the Trinity assemblies and Nanopore, the manually curated genes from the i5k effort, and the high confidence transcriptome-based gene models. As EvidenceModeler removes the UTR and alternative transcripts predicted from Augustus, we used PASA to update these models and after sanity checks we created the final JAMg OGS. The results were assessed using BUSCO (v 3.1.0).

Functional annotation of *B. oleae* gene models predicted by the JAMg annotation pipeline was performed using Blast2GO [45] included in OmicsBox version 1.1.78. Each protein (or the longest protein for multi-isoform genes) was Blast-searched again the Swiss Sprot database (1e-4) with output format 15 selected. XML Blastp results and sequences were imported into Blast2GO [45] and used to retrieve domain and motif signatures via Interproscan [46] analysis followed by identification of gene ontology (GO) terms via mapping and assignment of GO terms to sequences through functional annotation.

**Phylogenetic classification**

We used Prot-SpaM [47] to infer pairwise distances of 19 species using complete proteomes. A phylogenetic tree was generated using *Neighbour-Joining* algorithm [48] implemented in T-REX [49] and viewed using iTOL [50].

**Identification of orthologous proteins**

Orthologs among *D. melanogaster, M. domestica, C. capitata, Z. cucurbitae, B. dorsalis* and *B. oleae* were identified using OrthoFinder [51]. Supplementary Table S16 contains all orthogroups and the proteins from each species that belong to respective orthogroups.

**Principle Component Analysis and hierarchical clustering**

Gene expression (transcripts per million, TPM) was calculated for each of the 4 metamorphotic stages; egg, larvae, pupae, adult using RSEM [52] and used to calculate gene z-score on the log transformed TPM. Principle component analysis on the 1100 topmost variable genes among the stages was performed by the “prcomp” function then plotted by the “biplot” function both of R statistical software.

**Temporal clustering of developmental stage-specific genes**

The expression matrix (transcripts per million, TPM) filtered for genes that were not expressed at any of the stages was used as input to DPGP [53] to cluster genes with similar expression profiles. Clusters of Genes in clusters that peak at either of the 4 metamorphotic stages; egg, larvae, pupae, adult were combined and used in gene ontology enrichment analysis using gProfiler [54].

**References**

1. Tzanakakis ME, Economopoulos AP and Tsitsipis JA. The importance of conditions during the adult stage in evaluating an artificial food larvae of Dacus oleae (Gmelin) (Diptera: Tephritidae). Zeitschrift für Angewandte Entomologie. 1967;59 1-4:127-30. doi:10.1111/j.1439-0418.1967.tb03846.x.

2. Zhang M, Zhang Y, Scheuring CF, Wu CC, Dong JJ and Zhang HB. Preparation of megabase-sized DNA from a variety of organisms using the nuclei method for advanced genomics research. Nature protocols. 2012;7 3:467-78. doi:10.1038/nprot.2011.455.

3. Weisenfeld NI, Kumar V, Shah P, Church DM and Jaffe DB. Direct determination of diploid genome sequences. Genome Res. 2017;27 5:757-67. doi:10.1101/gr.214874.116.

4. Sagri E, Reczko M, Gregoriou ME, Tsoumani KT, Zygouridis NE, Salpea KD, et al. Olive fly transcriptomics analysis implicates energy metabolism genes in spinosad resistance. BMC genomics. 2014;15:714. doi:10.1186/1471-2164-15-714.

5. Bolger AM, Lohse M and Usadel B. Trimmomatic: a flexible trimmer for Illumina sequence data. Bioinformatics (Oxford, England). 2014;30 15:2114-20. doi:10.1093/bioinformatics/btu170.

6. Vurture GW, Sedlazeck FJ, Nattestad M, Underwood CJ, Fang H, Gurtowski J, et al. GenomeScope: fast reference-free genome profiling from short reads. Bioinformatics (Oxford, England). 2017;33 14:2202-4. doi:10.1093/bioinformatics/btx153.

7. Boisvert S, Raymond F, Godzaridis E, Laviolette F and Corbeil J. Ray Meta: scalable de novo metagenome assembly and profiling. Genome biology. 2012;13 12:R122. doi:10.1186/gb-2012-13-12-r122.

8. Chikhi R and Medvedev P. Informed and automated k-mer size selection for genome assembly. Bioinformatics (Oxford, England). 2014;30 1:31-7. doi:10.1093/bioinformatics/btt310.

9. Leggett RM, Clavijo BJ, Clissold L, Clark MD and Caccamo M. NextClip: an analysis and read preparation tool for Nextera Long Mate Pair libraries. Bioinformatics (Oxford, England). 2014;30 4:566-8. doi:10.1093/bioinformatics/btt702.

10. Li H. Aligning sequence reads, clone sequences and assembly contigs with BWA-MEM. ARXIV. 2013;00 00.

11. Boetzer M, Henkel CV, Jansen HJ, Butler D and Pirovano W. Scaffolding pre-assembled contigs using SSPACE. Bioinformatics (Oxford, England). 2011;27 4:578-9. doi:10.1093/bioinformatics/btq683.

12. Gurevich A, Saveliev V, Vyahhi N and Tesler G. QUAST: quality assessment tool for genome assemblies. Bioinformatics (Oxford, England). 2013;29 8:1072-5. doi:10.1093/bioinformatics/btt086.

13. Leggett RM, Heavens D, Caccamo M, Clark MD and Davey RP. NanoOK: multi-reference alignment analysis of nanopore sequencing data, quality and error profiles. Bioinformatics (Oxford, England). 2016;32 1:142-4. doi:10.1093/bioinformatics/btv540.

14. Koren S, Walenz BP, Berlin K, Miller JR, Bergman NH and Phillippy AM. Canu: scalable and accurate long-read assembly via adaptive k-mer weighting and repeat separation. Genome Research. 2017;27 5:722-36. doi:10.1101/gr.215087.116.

15. Tsoumani KT and Mathiopoulos KD. Genome size estimation with quantitative real-time PCR in two Tephritidae species: Ceratitis capitata and Bactrocera oleae. Journal of Applied Entomology. 2012;136 8:626-31. doi:10.1111/j.1439-0418.2011.01684.x.

16. Cao MD, Nguyen SH, Ganesamoorthy D, Elliott AG, Cooper MA and Coin LJ. Scaffolding and completing genome assemblies in real-time with nanopore sequencing. Nature communications. 2017;8:14515. doi:10.1038/ncomms14515.

17. Walker BJ, Abeel T, Shea T, Priest M, Abouelliel A, Sakthikumar S, et al. Pilon: An Integrated Tool for Comprehensive Microbial Variant Detection and Genome Assembly Improvement. PLoS ONE. 2014;9 11:e112963. doi:10.1371/journal.pone.0112963.

18. Kurtz S, Phillippy A, Delcher AL, Smoot M, Shumway M, Antonescu C, et al. Versatile and open software for comparing large genomes. Genome biology. 2004;5 2:R12. doi:10.1186/gb-2004-5-2-r12.

19. Simao FA, Waterhouse RM, Ioannidis P, Kriventseva EV and Zdobnov EM. BUSCO: assessing genome assembly and annotation completeness with single-copy orthologs. Bioinformatics (Oxford, England). 2015;31 19:3210-2. doi:10.1093/bioinformatics/btv351.

20. Hall AB, Qi Y, Timoshevskiy V, Sharakhova MV, Sharakhov IV and Tu Z. Six novel Y chromosome genes in Anopheles mosquitoes discovered by independently sequencing males and females. BMC genomics. 2013;14:273. doi:10.1186/1471-2164-14-273.

21. Drosopoulou E, Nakou I, Sichova J, Kubickova S, Marec F and Mavragani-Tsipidou P. Sex chromosomes and associated rDNA form a heterochromatic network in the polytene nuclei of Bactrocera oleae (Diptera: Tephritidae). Genetica. 2012;140 4-6:169-80. doi:10.1007/s10709-012-9668-3.

22. Mavragani-Tsipidou P. ZA, Drosopoulou E. A, A.A., Bourtzis K. MF. Tephritid Fruit Flies (Diptera). In: V. SI, editor. Protocols for Cytogenetic Mapping of Arthropod Genomes. Boca Raton, Florida: CRC Press; 2014. p. 1-60.

23. Mavragani-Tsipidou P, Karamanlidou G, Zacharopoulou A, Koliais S and Kastritisis C. Mitotic and polytene chromosome analysis in Dacus oleae (Diptera: Tephritidae). Genome. 1992;35 3:373-8.

24. Zambetaki A, Kleanthous K and Mavragani-Tsipidou P. Cytogenetic analysis of Malpighian tubule and salivary gland polytene chromosomes of Bactrocera oleae (Dacus oleae) (Diptera: Tephritidae). Genome. 1995;38 6:1070-81. doi:10.1139/g95-143.

25. Berthelier J, Casse N, Daccord N, Jamilloux V, Saint-Jean B and Carrier G. A transposable element annotation pipeline and expression analysis reveal potentially active elements in the microalga Tisochrysis lutea. BMC genomics. 2018;19 1:378. doi:10.1186/s12864-018-4763-1.

26. Smit AF, Hubley, R., Green, P.: RepeatMasker. http://www.repeatmasker.org (1996).

27. Han Y and Wessler SR. MITE-Hunter: a program for discovering miniature inverted-repeat transposable elements from genomic sequences. Nucleic acids research. 2010;38 22:e199. doi:10.1093/nar/gkq862.

28. Yang L and Bennetzen JL. Structure-based discovery and description of plant and animal Helitrons. Proceedings of the National Academy of Sciences of the United States of America. 2009;106 31:12832-7. doi:10.1073/pnas.0905563106.

29. Ellinghaus D, Kurtz S and Willhoeft U. LTRharvest, an efficient and flexible software for de novo detection of LTR retrotransposons. BMC bioinformatics. 2008;9 1:18. doi:10.1186/1471-2105-9-18.

30. Wenke T, Dobel T, Sorensen TR, Junghans H, Weisshaar B and Schmidt T. Targeted identification of short interspersed nuclear element families shows their widespread existence and extreme heterogeneity in plant genomes. The Plant cell. 2011;23 9:3117-28. doi:10.1105/tpc.111.088682.

31. Rho M, Choi JH, Kim S, Lynch M and Tang H. De novo identification of LTR retrotransposons in eukaryotic genomes. BMC genomics. 2007;8:90. doi:10.1186/1471-2164-8-90.

32. Hoede C, Arnoux S, Moisset M, Chaumier T, Inizan O, Jamilloux V, et al. PASTEC: an automatic transposable element classification tool. PLoS One. 2014;9 5:e91929. doi:10.1371/journal.pone.0091929.

33. Price AL, Jones NC and Pevzner PA. De novo identification of repeat families in large genomes. Bioinformatics (Oxford, England). 2005;21 Suppl 1:i351-8. doi:10.1093/bioinformatics/bti1018.

34. Goubert C, Modolo L, Vieira C, ValienteMoro C, Mavingui P and Boulesteix M. De novo assembly and annotation of the Asian tiger mosquito (Aedes albopictus) repeatome with dnaPipeTE from raw genomic reads and comparative analysis with the yellow fever mosquito (Aedes aegypti). Genome biology and evolution. 2015;7 4:1192-205. doi:10.1093/gbe/evv050.

35. Koch P, Platzer M and Downie BR. RepARK--de novo creation of repeat libraries from whole-genome NGS reads. Nucleic acids research. 2014;42 9:e80. doi:10.1093/nar/gku210.

36. Novak P, Neumann P, Pech J, Steinhaisl J and Macas J. RepeatExplorer: a Galaxy-based web server for genome-wide characterization of eukaryotic repetitive elements from next-generation sequence reads. Bioinformatics (Oxford, England). 2013;29 6:792-3. doi:10.1093/bioinformatics/btt054.

37. Weizhong L: CD-HIT-EST. http://weizhongli-lab.org/cd-hit/ (2006).

38. Flutre T, Duprat E, Feuillet C and Quesneville H. Considering transposable element diversification in de novo annotation approaches. PLoS One. 2011;6 1:e16526. doi:10.1371/journal.pone.0016526.

39. Haas BJ, Papanicolaou A, Yassour M, Grabherr M, Blood PD, Bowden J, et al. De novo transcript sequence reconstruction from RNA-seq using the Trinity platform for reference generation and analysis. Nature protocols. 2013;8 8:1494-512. doi:10.1038/nprot.2013.084.

40. Grabherr MG, Haas BJ, Yassour M, Levin JZ, Thompson DA, Amit I, et al. Full-length transcriptome assembly from RNA-Seq data without a reference genome. Nature biotechnology. 2011;29 7:644-52. doi:10.1038/nbt.1883.

41. C. Titus Brown AH, Qingpeng Zhang, Alexis B. Pyrkosz, Timothy H. Brom. A Reference-Free Algorithm for Computational Normalization of Shotgun Sequencing Data. ARXIV. 2012;1203.4802v2 [q-bio.GN].

42. Papanicolaou A: https://github.com/genomecuration/JAMg Accessed 19/Aug/2019.

43. Papanicolaou A, Schetelig MF, Arensburger P, Atkinson PW, Benoit JB, Bourtzis K, et al. The whole genome sequence of the Mediterranean fruit fly, Ceratitis capitata (Wiedemann), reveals insights into the biology and adaptive evolution of a highly invasive pest species. Genome biology. 2016;17 1:192. doi:10.1186/s13059-016-1049-2.

44. Haas BJ, Delcher AL, Mount SM, Wortman JR, Smith RK, Jr., Hannick LI, et al. Improving the Arabidopsis genome annotation using maximal transcript alignment assemblies. Nucleic acids research. 2003;31 19:5654-66. doi:10.1093/nar/gkg770.

45. Conesa A, Gotz S, Garcia-Gomez JM, Terol J, Talon M and Robles M. Blast2GO: a universal tool for annotation, visualization and analysis in functional genomics research. Bioinformatics (Oxford, England). 2005;21 18:3674-6. doi:10.1093/bioinformatics/bti610.

46. Jones P, Binns D, Chang HY, Fraser M, Li W, McAnulla C, et al. InterProScan 5: genome-scale protein function classification. Bioinformatics (Oxford, England). 2014;30 9:1236-40. doi:10.1093/bioinformatics/btu031.

47. Leimeister C-A, Schellhorn J, Schöbel S, Gerth M, Bleidorn C and Morgenstern B. &lt;em&gt;Prot-SpaM&lt;/em&gt;: Fast alignment-free phylogeny reconstruction based on whole-proteome sequences. bioRxiv. 2018:306142. doi:10.1101/306142.

48. Saitou N and Nei M. The neighbor-joining method: a new method for reconstructing phylogenetic trees. Molecular biology and evolution. 1987;4 4:406-25. doi:10.1093/oxfordjournals.molbev.a040454.

49. Boc A, Diallo AB and Makarenkov V. T-REX: a web server for inferring, validating and visualizing phylogenetic trees and networks. Nucleic acids research. 2012;40 Web Server issue:W573-9. doi:10.1093/nar/gks485.

50. Letunic I and Bork P. Interactive tree of life (iTOL) v3: an online tool for the display and annotation of phylogenetic and other trees. Nucleic acids research. 2016;44 W1:W242-5. doi:10.1093/nar/gkw290.

51. Emms DM and Kelly S. OrthoFinder: phylogenetic orthology inference for comparative genomics. bioRxiv. 2019:466201. doi:10.1101/466201.

52. Li B and Dewey CN. RSEM: accurate transcript quantification from RNA-Seq data with or without a reference genome. BMC bioinformatics. 2011;12:323. doi:10.1186/1471-2105-12-323.

53. McDowell IC, Manandhar D, Vockley CM, Schmid AK, Reddy TE and Engelhardt BE. Clustering gene expression time series data using an infinite Gaussian process mixture model. PLoS computational biology. 2018;14 1:e1005896. doi:10.1371/journal.pcbi.1005896.

54. Reimand J, Kull M, Peterson H, Hansen J and Vilo J. g:Profiler--a web-based toolset for functional profiling of gene lists from large-scale experiments. Nucleic acids research. 2007;35 Web Server issue:W193-200. doi:10.1093/nar/gkm226.

55. Carvalho AB, Clark AG: Efficient identification of Y chromosome sequences in the human and Drosophila genomes. Genome Res 2013, 23:1894-1907.
