# Supplementary material for: De novo assembly of the olive fruit fly (Bactrocera oleae) genome with linked-reads and long-read technologies minimizes gaps and provides exceptional Y chromosome assembly
Source: BMC Genomics. 2020 Mar 30;21:259. doi: 10.1186/s12864-020-6672-3 (PMC7106766; doi:10.1186/s12864-020-6672-3)
Supplement: Supplementary file 3 — Additional file 3: Figure S1. Schematic of the method used to generate the main assembly reported. Figure S2. Genome size and heterozygosity estimation. Figure S3. Contig length at different Nx values for assemblies in Supplementary Table S3. Figure S4. Contiguity plot generated using Quast. Figure S5. Contig length at different Nx values for assemblies in Supplementary Table S4. Figure S6. Plot showing Y chromosome scaffolds/contigs identified in 3 different assemblies (Supplementary Table S4). Figure S7. Total length of scaffolds that were localized to each polytene chromosome and XY chromosomes. Figure S8. Alignment rates of RNA-seq reads from 12 different Bactrocera oleae datasets (see Supplementary Table S9). Figure S9. Complete Basic Universal Single Copy Orthologs (BUSCOs) identified in genome assemblies (Supplementary Table S3). Figure S10. Schematic of the PiRATE pipeline. Figure S11. Histogram of transcripts read lengths. Figure S12. Percentage of Arthropoda Basic Universal Single Copy Orthologs (BUSCOs) captured in 19 arthropod transcriptomes. Figure S13. Number of JAMg predicted B. oleae genes located on the scaffolds assigned to polytene element. Figure S14. Gene ontology (GO) classification of B. oleae JAMg predicted proteins. Figure S15. Detailed orthogroup distribution. Figure S16. Hierarchical clustering of 1100 most variable genes among the 4 metamorphotic stages. Figure S17. Most significantly enriched gene ontology (GO) terms among genes that only peak during development. Figure S18. Contig length at different Nx values for assemblies of selected insects. [file 12864_2020_6672_MOESM3_ESM.docx]

***De novo* assembly of the olive fruit fly (*Bactrocera oleae*) genome with linked-reads and long-read technologies minimizes gaps and provides exceptional Y chromosome assembly**


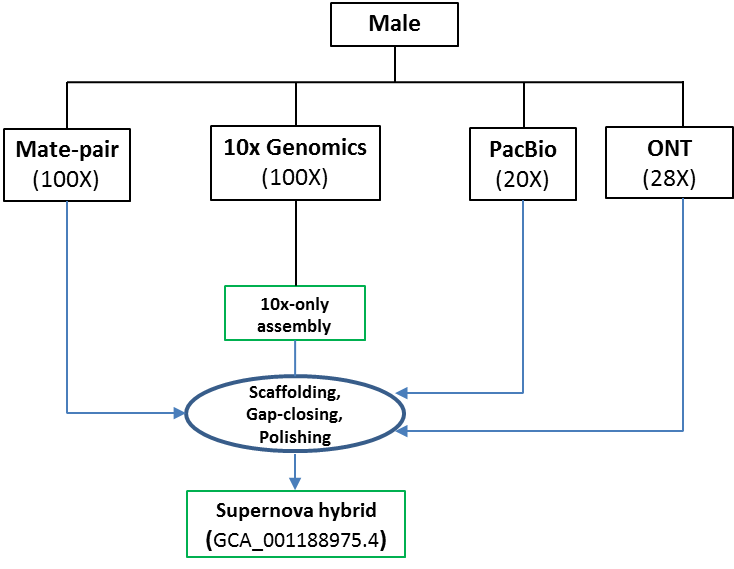


**Supplementary Figure S1: Schematic of the method used to generate the main assembly reported.** DNA extracted from adult male insects was used to generate and sequencing libraries for; Illumina mate-pair (MP) (20X coverage), 10x Genomics linked-reads (100X coverage generated but 74X was found optimal for genome assembly), Pacific Biosciences (PacBio, 20X coverage), and Oxford Nanopore Technologies (ONT, 28X coverage). A linked-reads based genome assembly was generated (10x-only assembly) which was scaffolded and gap-closed using mate-pair, PacBio, and ONT data and finally polished using Pilon and submitted to NCBI (GenBank accession GCA_001188975.4)


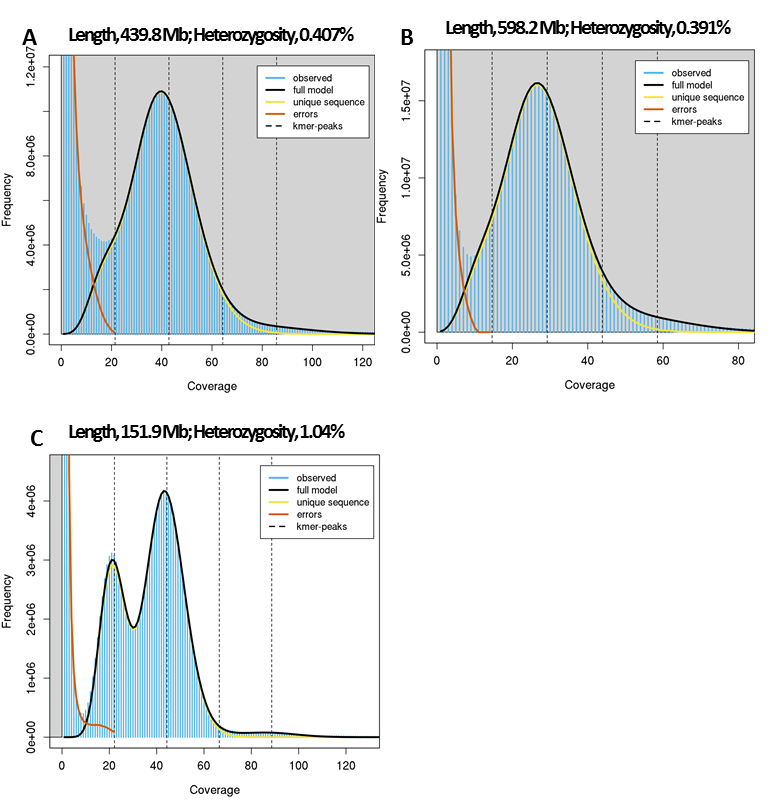


**Supplementary Figure S2: Genome size and heterozygosity estimation.** (A) *Bactrocera oleae*, (B) *Ceratitis capitata* and (C) *Arabidopsis thaliana* (included here to show profile of high heterozygosity). Estimates were generated using GenomeScope [1].


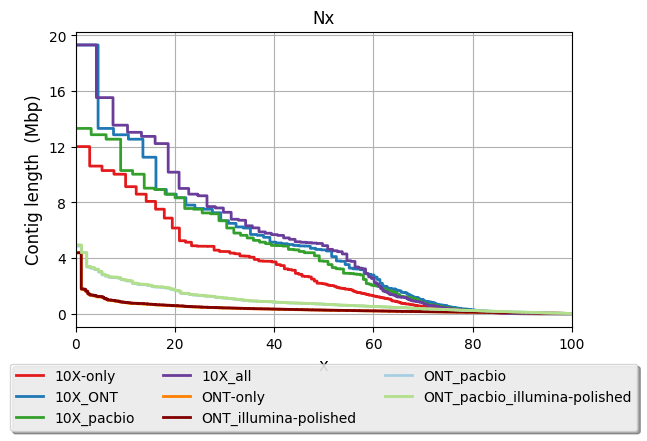


**Supplementary Figure S3: Contig length at different Nx values for assemblies in Supplementary Table S3.** Nx value is the scaffold/contig length at which x % of the genome is contained in scaffolds/contigs at or above that length. Graph was generated using Quast [2].


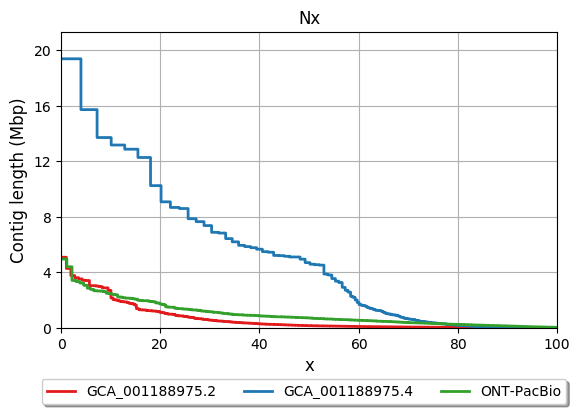


**Supplementary Figure S4: Contiguity plot generated using Quast [2].** Three assemblies were assessed; our initial assembly generated from Illumina short-read assembly followed by long-read gap-closing (GenBank assembly accession: GCA_001188975.2), an assembly generated from Oxford Nanopore technology and Pacific Biosciences long-read sequencing technologies (ONT-PacBio), and an assembly generated from the 10x Genomics linked-reads technology followed by scaffolding and gap-closing using Illumina mate-pair, ONT, and PacBio reads (GenBank assembly accession: GCA_001188975.4). Nx value is the scaffold/contig length at which x % of the genome is contained in scaffolds/contigs at or above that length.


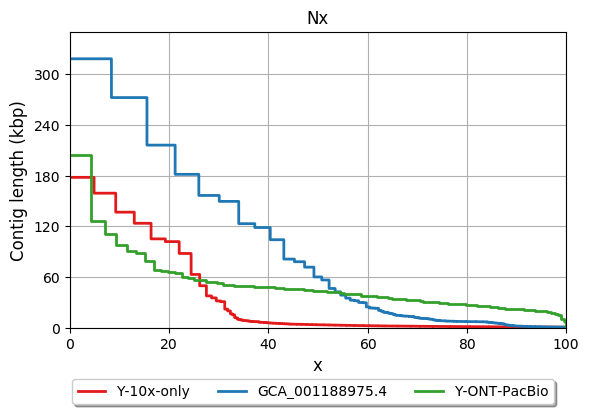


**Supplementary Figure S5: Contig length at different Nx values for assemblies in Supplementary Table S4**. Contiguity plot generated using Quast [2]. Nx value is the scaffold/contig length at which x % of the genome is contained in scaffolds/contigs at or above that length.


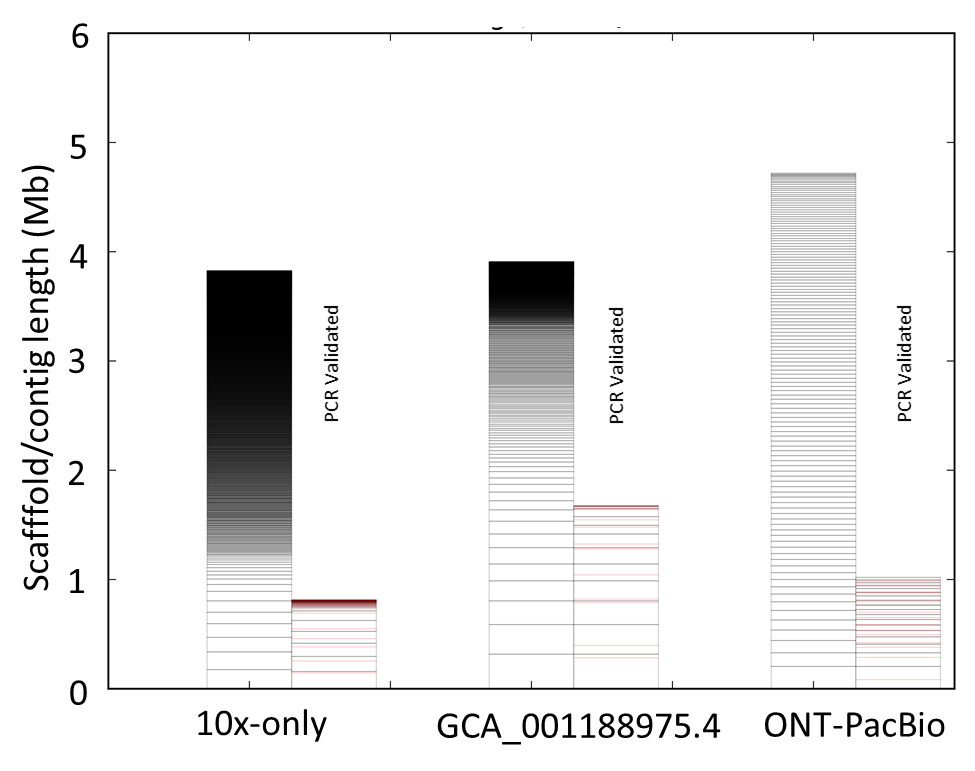


**Supplementary Figure S6: Plot showing Y chromosome scaffolds/contigs identified in 3 different assemblies (Supplementary Table S4).** The Chromosome Quotient (CQ) method [3] was used to identify Y chromosome scaffolds. The scaffolds/contigs are ordered from longest to shortest. For each assembly the total scaffolds/contigs are shown in left bars while the PCR validated scaffolds/contigs are the right bars. The approximate location of the PCR primer on the scaffold/contig is shown in pink.


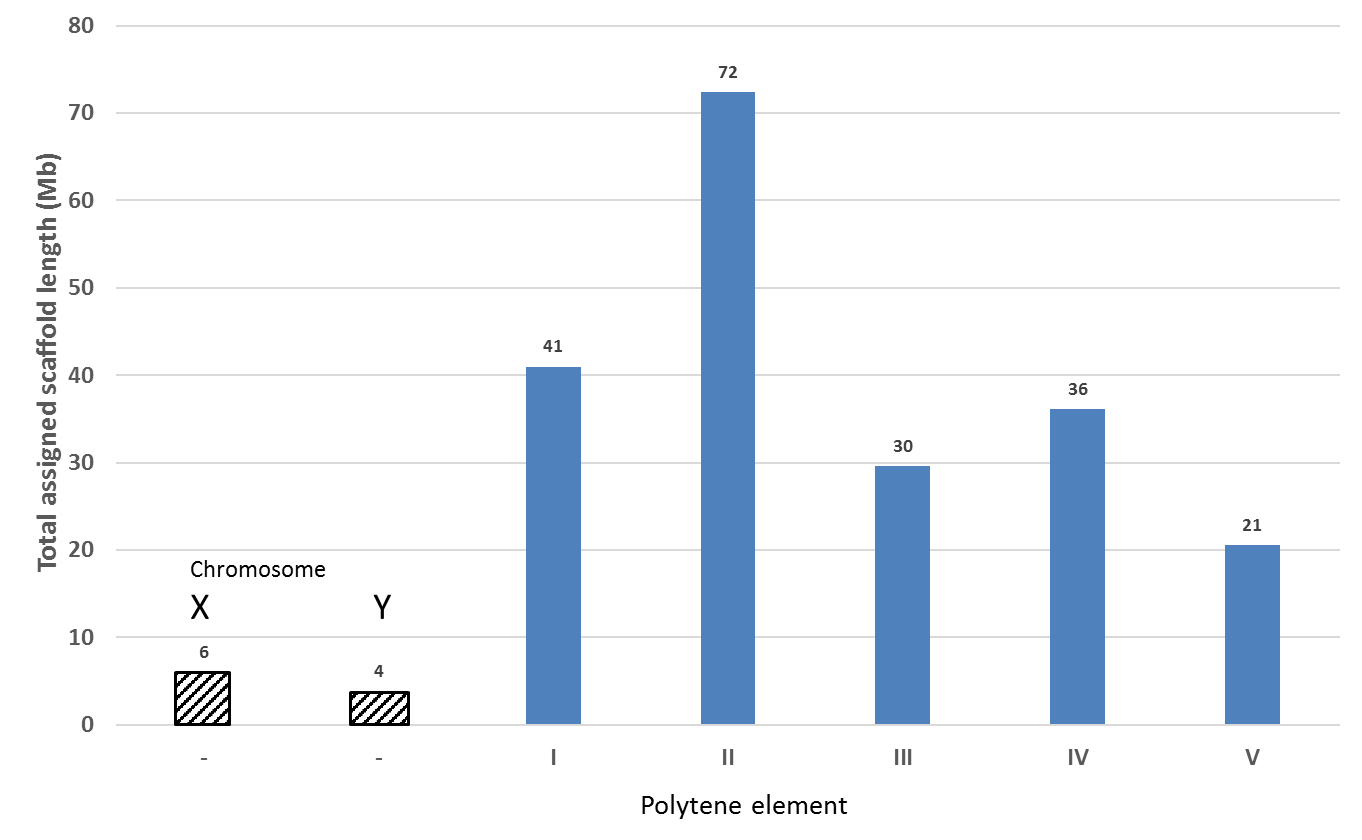


**Supplementary Figure S7: Total length of scaffolds that were localized to each polytene chromosome and XY chromosomes**. For polytene element I to V, markers detailed in Supplementary Table S7 and Supplementary Table S11 that were previously localized to polytene chromosomes were aligned to scaffolds. Scaffolds that bore these markers were assigned to the corresponding chromosomes. The X and Y chromosome scaffolds/contigs (shown with stripped bars) were identified using the Chromosome Quotient method [3]. Out of 4 Mb of the Y chromosome 1.7 Mb was validated using PCR.

**Supplementary Figure S8: Alignment rates of RNAseq reads from 15 different *Bactrocera oleae* datasets (see Supplementary Table S9).** GCA_001188975.2 Transcriptome, GCA_001188975.2 NCBI predicted official gene set (OGS); GCA_001188975.2 Genome, GCA_001188975.2 genome assembly; GCA_001188975.4 Transcriptome, GCA_001188975.4 JAMg OGS; GCA_001188975.4 Genome, GCA_001188975.4 genome; Trinity de novo, Trinity *de novo* transcriptome assembly; 10x Diploid Genome, Combined haplotypes from the Supernova *B. oleae* assembly. Read alignment and alignment rate computation were done using HISAT2 [4].


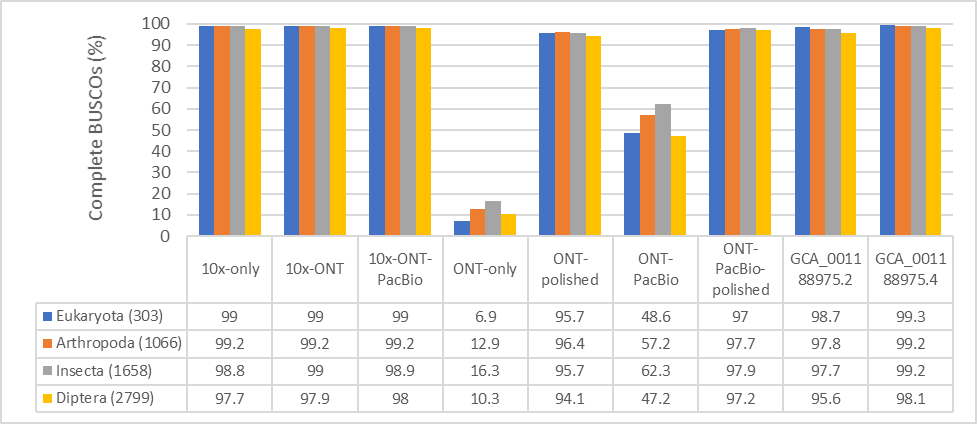


**Supplementary Figure S9: Complete Basic Universal Single Copy Orthologs (BUSCOs, [5]) identified in genome assemblies (Supplementary Table S3).** BUSCOs from Eukaryota (303), Arthropoda (1066), Insecta (1658) and Diptera (2799) were surveyed. Long-read assemblies ONT-only and ONT-PacBio were polished using short reads generated with Illumina sequencing.


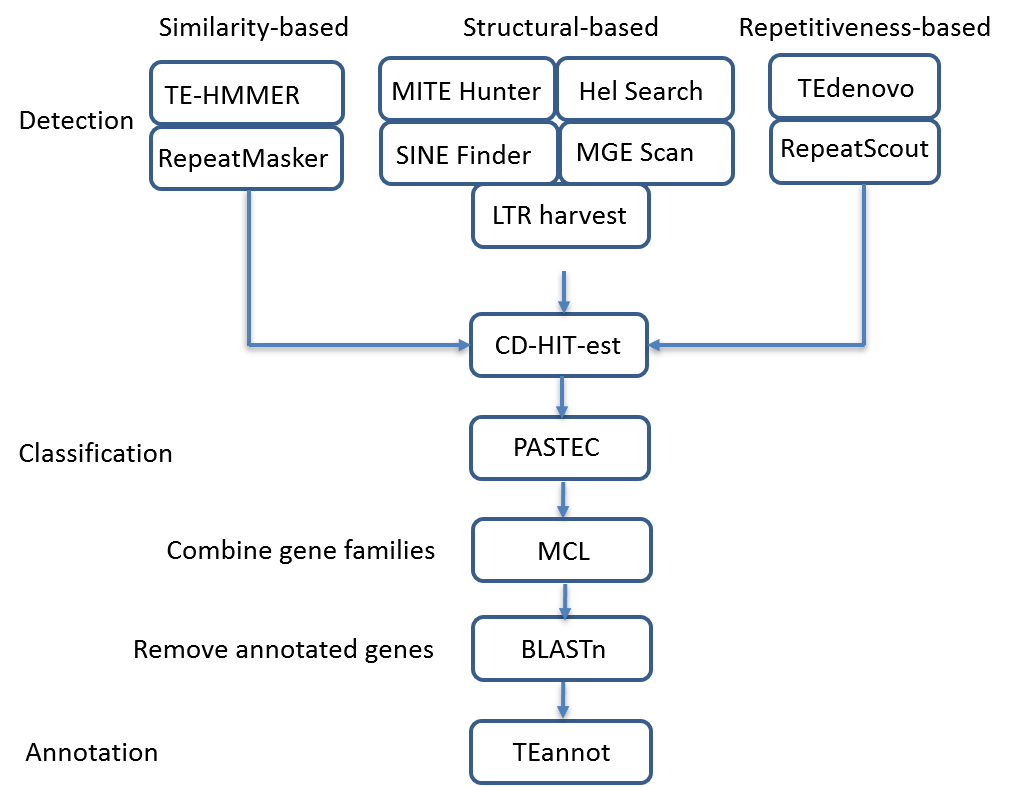


**Supplementary Figure S10: Schematic of the PiRATE pipeline [6].** PiRATE was used to identify transposable elements (TE) and annotate TE in the *Bactrocera oleae* genome. Nine genome based TE identification tools were used to identify TE in *B. oleae* genome. Overlapping TE were removed using CD-HIT-est and the TE classified using PASTEC [7]. The library was also BLAST’ed against the *B. oleae* proteome to exclude protein coding genes. Finally, the library was used to annotate the genome using TEannot [8].


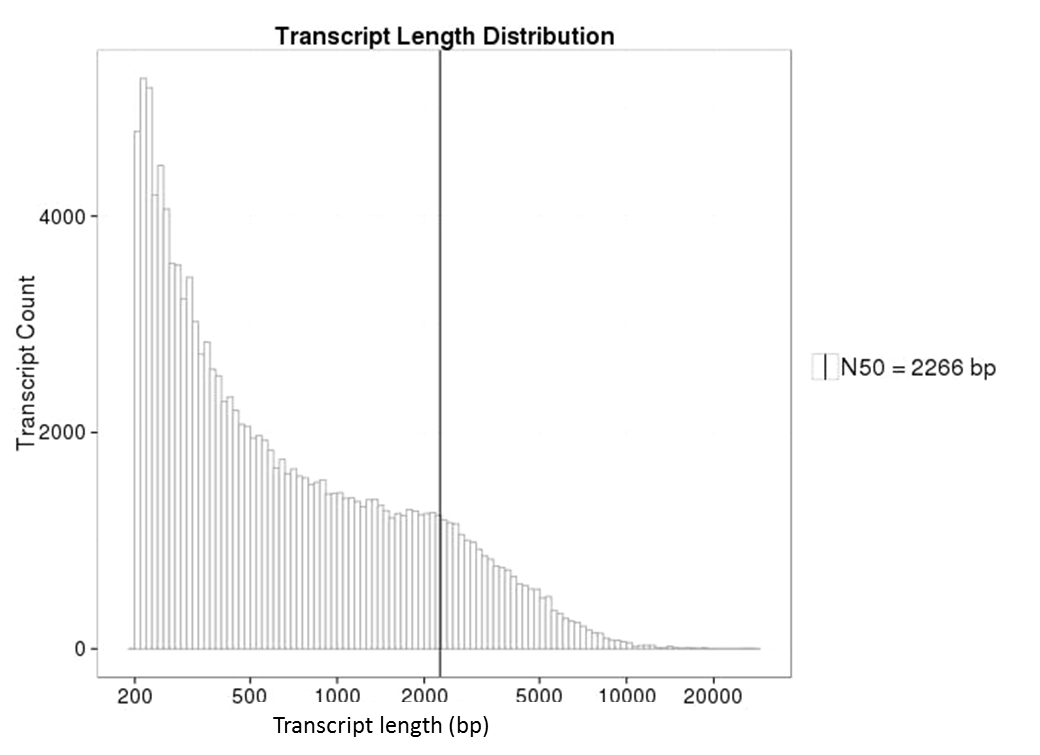


**Supplementary Figure S11: Histogram of transcripts read lengths.** Transcripts were assembled *de novo* using Trinity [9] using the datasets shown in Supplementary Table S10.

**Supplementary Figure S12: Percentage of Arthropoda Basic Universal Single Copy Orthologs (BUSCOs, [5]) captured in 19 arthropod transcriptomes**. The percentage of complete (single or duplicated), fragmented, and missing BUSCOs among 1066 surveyed is shown. Aae (*Aedes aegypti*, yellow fever mosquito), Aga (*Anopheles gambiae*, African malaria mosquito), Ame (*Acyrthosiphon pisum*, pea aphid), Api (*Apis mellifera*, honey bee), Bdo (*Bactrocera dorsalis*, oriental fruit fly), Bim (*Bombus impatiens*, common eastern bumble bee), Bmo (Bombyx mori, domestic silkworm), Bol (*Bactrocera oleae,* olive fruit fly), Cca (*Ceratitis capitata*, Mediterranean fruit fly), Cle (*Cimex lectularius*, Bed bug), Cqu (*Culex quinquefasciatus*, southern house mosquito), Dme (*Droshophila melanogaster*, Fruit fly), Dpu (*Daphnia pulex*, common water flea), Mdo (*Musca domestica*, House fly), Mse (*Manduca sexta*, tobacco hornworm), Phu (*Pediculus humanus*, human louse), Sin (*Solenopsis invicta*, Red fire ant), Tca (*Tribolium castaneum*, Red flour beetle), Zcu (*Zeugodacus cucurbitae*, melon fly). See Supplementary Table S16 for sources of the proteomes used.

**Supplementary Figure S13: Number of JAMg predicted *B. oleae* genes located on the scaffolds assigned to polytene element**. “Un” refers to genes located on scaffolds/contigs that are not yet assigned to any polytene element.


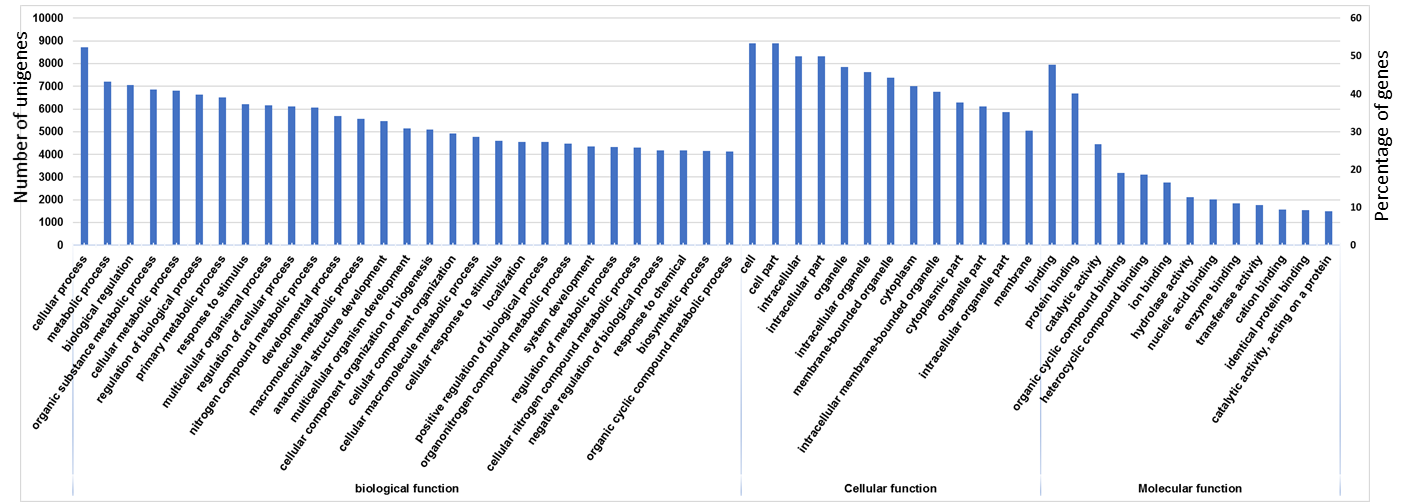


**Supplementary Figure S14: Gene ontology (GO) classification of *B. oleae* JAMg predicted proteins**. Top GO annotations in each of the main groups are shown. The annotation was performed with BLAST2GO [10].


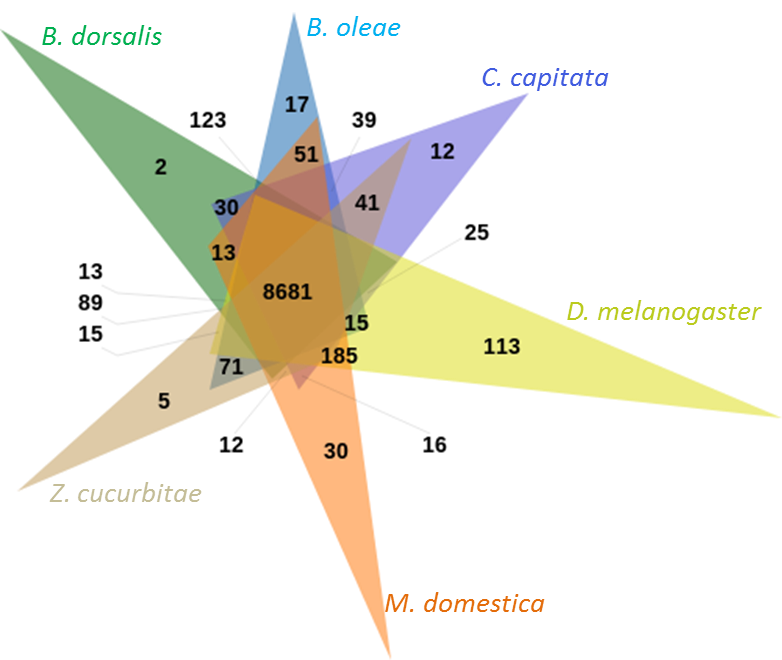


**Supplementary Figure S15: Detailed orthogroup distribution**. Venn diagram of shared orthogroups among 6 closely related dipteran insects; *B. oleae, C. capitata, D. melanogaster, Z. cucurbitae, B. dorsalis* and *M. domestica.* Orthologous proteins were identified and grouped using OrthoFinder [11]. Shared and unique orthogroups are plotted using Jvenn [12]. See Supplementary Table S16 for sources of the proteomes used.


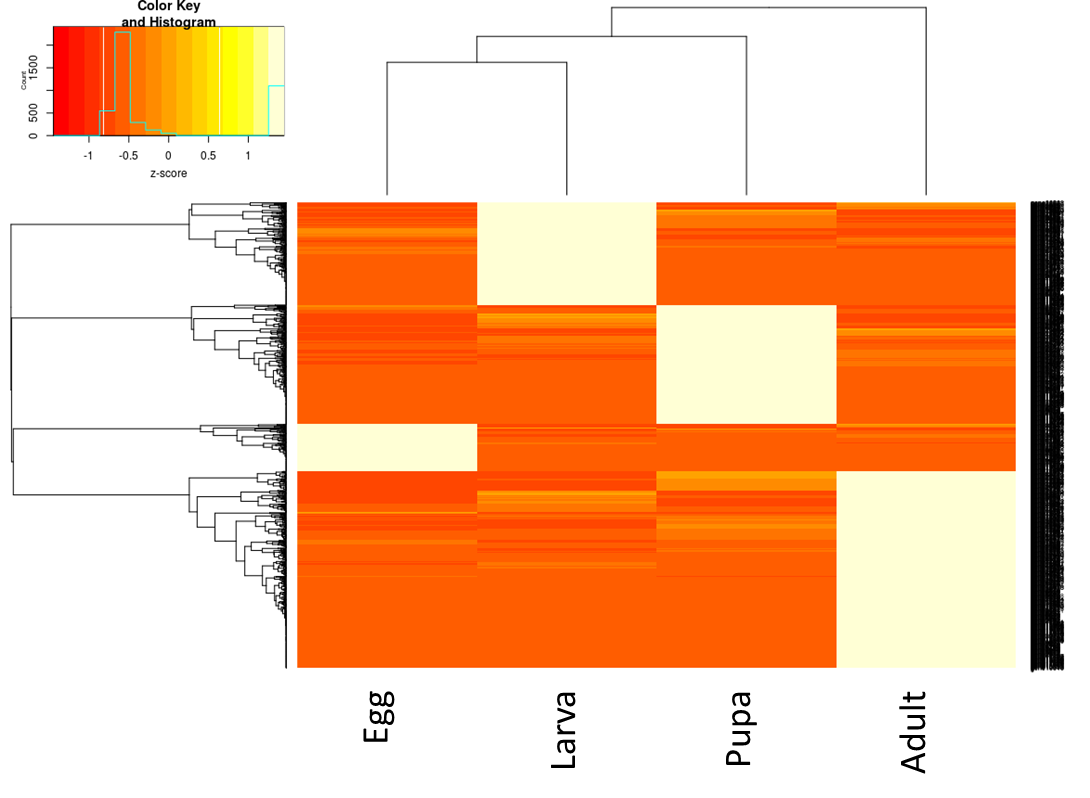


**Supplementary Figure S16: Hierarchical clustering of 1100 most variable genes among the 4 metamorphotic stages**. Gene expression (transcripts per million, TPM) was calculated for each of the stages; egg, larvae, pupae, and adult using RSEM [13] and used to calculate gene z-score on the log transformed TPM.


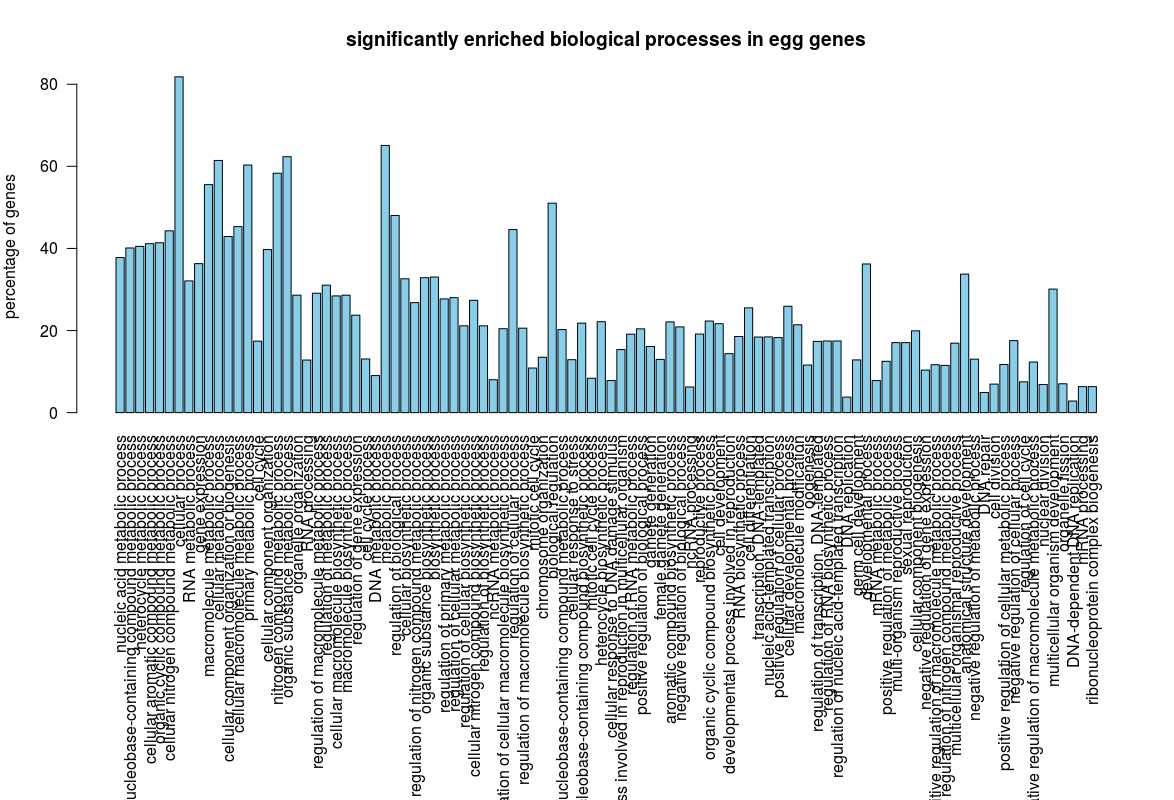


**Supplementary Figure S17 (A): Most significantly enriched gene ontology (GO) terms among genes that only peak during egg development**. The genes were determined using DPGP [14] and the corresponding *D. melanogaster* homologs determined using BLASTp. Significantly enriched GO terms associated with corresponding Uniprot protein IDs were identified using gProfiler [15].


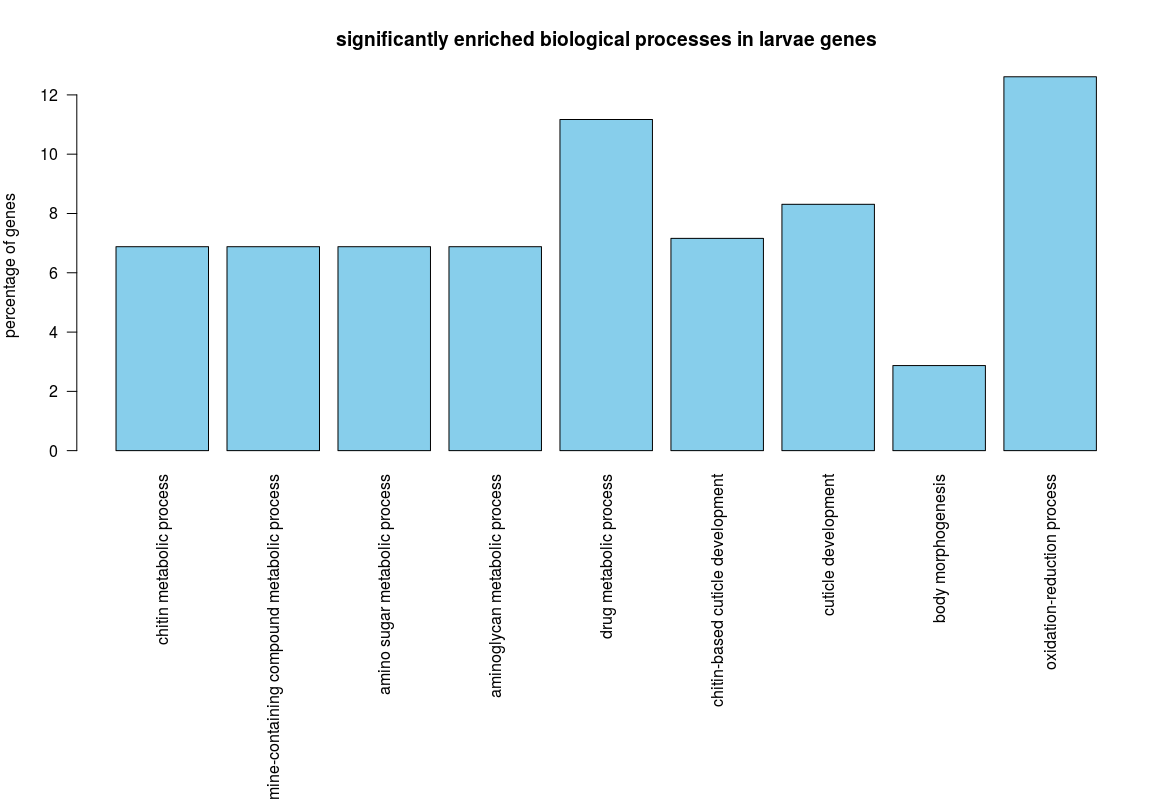


**Supplementary Figure S17 B): Most significantly enriched gene ontology (GO) terms among genes that only peak during larvae development**. The genes were determined using DPGP [14] and the corresponding *D. melanogaster* homologs determined using BLASTp. Significantly enriched GO terms associated with corresponding Uniprot protein IDs were identified using gProfiler [15].


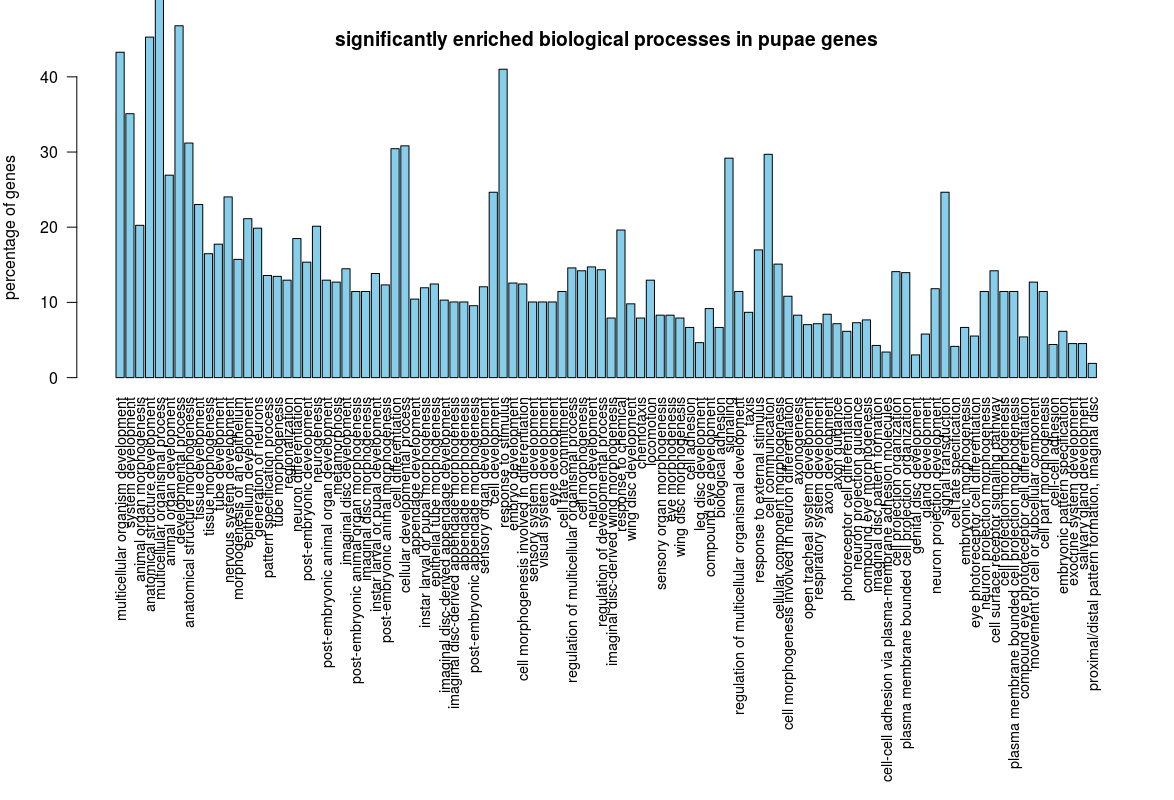


**Supplementary Figure S17 C): Most significantly enriched gene ontology (GO) terms among genes that only peak during pupae development**. The genes were determined using DPGP [14] and the corresponding *D. melanogaster* homologs determined using BLASTp. Significantly enriched GO terms associated with corresponding Uniprot protein IDs were identified using gProfiler [15].


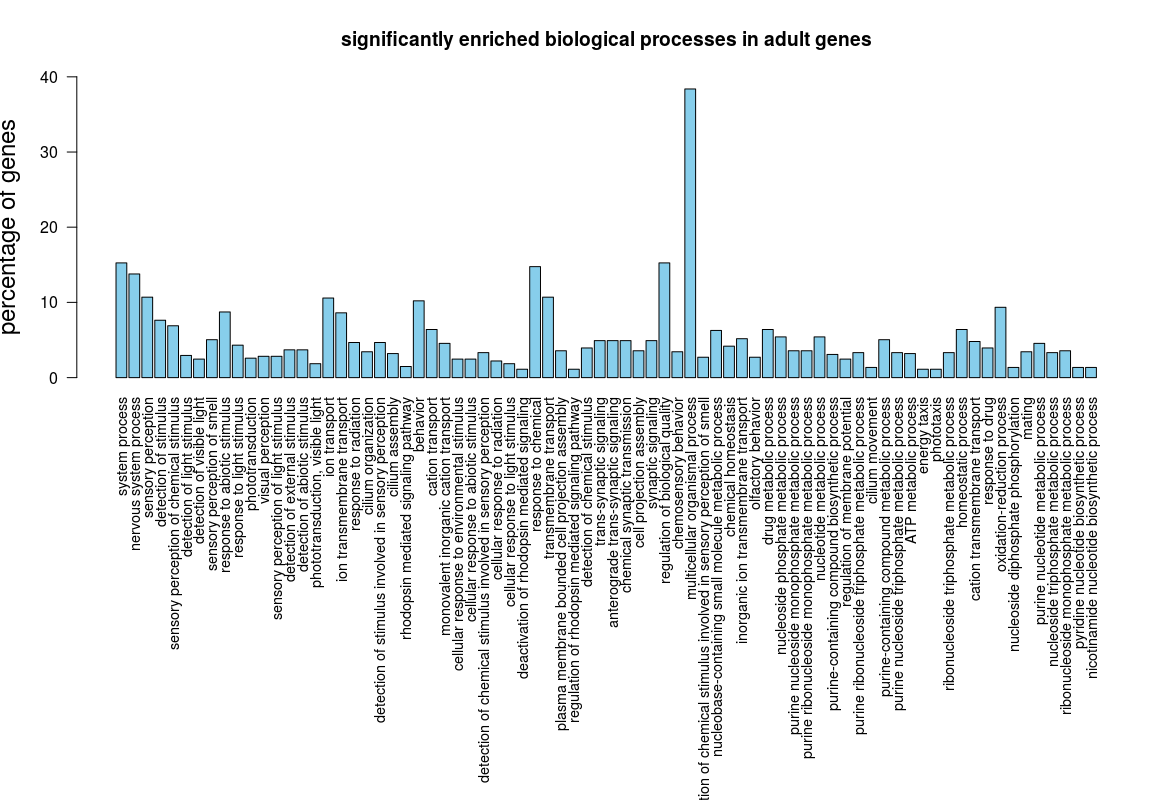


**Supplementary Figure S17 D): Most significantly enriched gene ontology (GO) terms among genes that only peak during adult development**. The genes were determined using DPGP [14] and the corresponding *D. melanogaster* homologs determined using BLASTp. Significantly enriched GO terms associated with corresponding Uniprot protein IDs were identified using gProfiler [15].


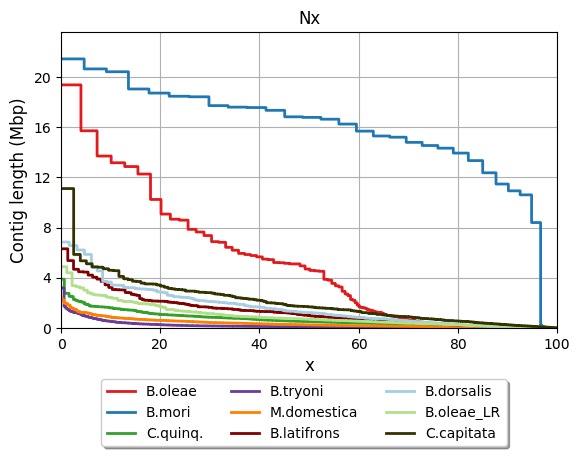


**Supplementary Figure S18: Contig length at different Nx values for assemblies of selected insects**. B.oleae, *Bactrocera oleae* GCA_001188975.4 assembly; B.oleae_LR, *Bactrocera oleae* ONT-PacBio long-read assembly; B.mori, *Bombyx mori*; C.quinq., *Culex quinquefasciatus*; B.tyroni, *Bactrocera tyroni*; M.domestica, *Musca domestica*; B.latifrons, *Bactrocera latifrons*; B.dorsalis, *Bactrocera dorsalis*; C.capitata, *Ceratitis capitata*. Contiguity plot generated using Quast [2]. Nx value is the scaffold/contig length at which x % of the genome is contained in scaffolds/contigs at or above that length. The y-axis shows the contig/scaffold length at each of the Nx values. See Supplementary Table S15 for sources of the genomes used.

**References**

1. Vurture GW, Sedlazeck FJ, Nattestad M, Underwood CJ, Fang H, Gurtowski J, Schatz MC: **GenomeScope: fast reference-free genome profiling from short reads.** *Bioinformatics* 2017, **33:**2202-2204.

2. Gurevich A, Saveliev V, Vyahhi N, Tesler G: **QUAST: quality assessment tool for genome assemblies.** *Bioinformatics* 2013, **29:**1072-1075.

3. Hall AB, Qi Y, Timoshevskiy V, Sharakhova MV, Sharakhov IV, Tu Z: **Six novel Y chromosome genes in Anopheles mosquitoes discovered by independently sequencing males and females.** *BMC Genomics* 2013, **14:**273.

4. Kim D, Paggi JM, Park C, Bennett C, Salzberg SL: **Graph-based genome alignment and genotyping with HISAT2 and HISAT-genotype.** *Nat Biotechnol* 2019, **37:**907-915.

5. Simao FA, Waterhouse RM, Ioannidis P, Kriventseva EV, Zdobnov EM: **BUSCO: assessing genome assembly and annotation completeness with single-copy orthologs.** *Bioinformatics* 2015, **31:**3210-3212.

6. Berthelier J, Casse N, Daccord N, Jamilloux V, Saint-Jean B, Carrier G: **A transposable element annotation pipeline and expression analysis reveal potentially active elements in the microalga Tisochrysis lutea.** *BMC Genomics* 2018, **19:**378.

7. Hoede C, Arnoux S, Moisset M, Chaumier T, Inizan O, Jamilloux V, Quesneville H: **PASTEC: an automatic transposable element classification tool.** *PLoS One* 2014, **9:**e91929.

8. Flutre T, Duprat E, Feuillet C, Quesneville H: **Considering transposable element diversification in de novo annotation approaches.** *PLoS One* 2011, **6:**e16526.

9. Grabherr MG, Haas BJ, Yassour M, Levin JZ, Thompson DA, Amit I, Adiconis X, Fan L, Raychowdhury R, Zeng Q, et al: **Full-length transcriptome assembly from RNA-Seq data without a reference genome.** *Nat Biotechnol* 2011, **29:**644-652.

10. Conesa A, Gotz S, Garcia-Gomez JM, Terol J, Talon M, Robles M: **Blast2GO: a universal tool for annotation, visualization and analysis in functional genomics research.** *Bioinformatics* 2005, **21:**3674-3676.

11. Emms DM, Kelly S: **OrthoFinder: phylogenetic orthology inference for comparative genomics.** *bioRxiv* 2019**:**466201.

12. Bardou P, Mariette J, Escudie F, Djemiel C, Klopp C: **jvenn: an interactive Venn diagram viewer.** *BMC Bioinformatics* 2014, **15:**293.

13. Li B, Dewey CN: **RSEM: accurate transcript quantification from RNA-Seq data with or without a reference genome.** *BMC Bioinformatics* 2011, **12:**323.

14. McDowell IC, Manandhar D, Vockley CM, Schmid AK, Reddy TE, Engelhardt BE: **Clustering gene expression time series data using an infinite Gaussian process mixture model.** *PLoS Comput Biol* 2018, **14:**e1005896.

15. Reimand J, Kull M, Peterson H, Hansen J, Vilo J: **g:Profiler--a web-based toolset for functional profiling of gene lists from large-scale experiments.** *Nucleic Acids Res* 2007, **35:**W193-200.
